# Transient Astrocytic Gq Signaling Underlies Remote Memory Enhancement

**DOI:** 10.3389/fncir.2021.658343

**Published:** 2021-03-22

**Authors:** Youichi Iwai, Katsuya Ozawa, Kazuko Yahagi, Tsuneko Mishima, Sonam Akther, Camilla Trang Vo, Ashley Bomin Lee, Mika Tanaka, Shigeyoshi Itohara, Hajime Hirase

**Affiliations:** ^1^Laboratory for Neuron-Glia Circuitry, RIKEN Center for Brain Science, Wako, Japan; ^2^Center for Translational Neuromedicine, Faculty of Medical and Health Sciences, University of Copenhagen, Copenhagen, Denmark; ^3^Department of Biology, Faculty of Science, University of Copenhagen, Copenhagen, Denmark; ^4^Laboratory for Behavioral Genetics, RIKEN Center for Brain Science, Wako, Japan

**Keywords:** astrocytes, optogenetic activation, G protein-coupled receptor, Ca^2+^ signaling, memory, OptoA1AR

## Abstract

Astrocytes elicit transient Ca^2+^ elevations induced by G protein-coupled receptors (GPCRs), yet their role *in vivo* remains unknown. To address this, transgenic mice with astrocytic expression of the optogenetic Gq-type GPCR, Optoα1AR, were established, in which transient Ca^2+^ elevations similar to those in wild type mice were induced by brief blue light illumination. Activation of cortical astrocytes resulted in an adenosine A1 receptor-dependent inhibition of neuronal activity. Moreover, sensory stimulation with astrocytic activation induced long-term depression of sensory evoked response. At the behavioral level, repeated astrocytic activation in the anterior cortex gradually affected novel open field exploratory behavior, and remote memory was enhanced in a novel object recognition task. These effects were blocked by A1 receptor antagonism. Together, we demonstrate that GPCR-triggered Ca^2+^ elevation in cortical astrocytes has causal impacts on neuronal activity and behavior.

## Introduction

Interfacing both synapses and blood vessels, astrocytes' prime functions in the brain have been recognized as the maintenance of extracellular environment and the transfer of energy substrates. Additionally, studies in the recent decade have presented compelling evidence that astrocytes modulate neuronal activity by various mechanisms. Astrocytes sense neural activities and induce intracellular signaling *via* multiple ions and second messengers including Ca^2+^, Na^2+^, H^+^, and cAMP (Verkhratsky et al., 2020, for a review). Among those, the most pronounced concentration changes occur with Ca^2+^. While electrically passive, astrocytes elicit large-amplitude cytosolic Ca^2+^ elevations that are triggered by G protein-coupled receptors (GPCRs), particularly the Gq-type, which activate the inositol trisphosphate (IP_3_) pathway. Amongst Gq-GPCRs, the alpha-1 adrenergic receptor (α1AR) has been identified to be the prevalent receptor for brain-wide astrocytic Ca^2+^ elevations, responding to locus coeruleus (LC) activation in awake mice (Ding et al., 2013; Paukert et al., 2014).

A considerable amount of literature suggests modulation of synaptic transmission and plasticity by astrocytic Ca^2+^ elevation (Fellin et al., 2004; Fiacco and McCarthy, 2004; Jourdain et al., 2007; Henneberger et al., 2010; Di Castro et al., 2011; Panatier et al., 2011; Takata et al., 2011; Navarrete et al., 2012; Monai et al., 2016; Pinto-Duarte et al., 2019). On the other hand, there are a few studies that report negative results (Petravicz et al., 2008; Agulhon et al., 2010). Moreover, a study that performed a behavioral test battery reported no obvious phenotype in astrocyte-specific IP_3_R2-KO mice (Petravicz et al., 2014). Recently, however, the use of synthetic GPCRs (i.e., DREADDs) permitted astrocyte-specific pharmacogenetic activation of Gq signaling. While an initial study that targeted brain-wide astrocytes did not find a phenotype in motor learning (Agulhon et al., 2013), recent studies reported phenotypes in aversive learning by amygdalar or hippocampal astrocytic activation (Martin-Fernandez et al., 2017; Adamsky et al., 2018). However, pharmacogenetic activation of astrocytes inevitably leads to hour-long activation of astrocytes, hence the role of physiological activation of astrocytes in behaving mice has remained unaddressed. Here, we generated transgenic (TG) mouse lines in which astrocytes express the optogenetically activated Gq-GPCR Optoα1AR (Airan et al., 2009) that permits transient elevation of astrocytic Ca^2+^ by blue light in inflammation-free conditions.

## Materials and Methods

The animal study was reviewed and approved by the RIKEN Institutional Animal Care and Use Committee and the Danish Animal Experiments Inspectorate.

### Generation of Transgenic Mice

The PCR fragment of *Opto*α*1AR-EYFP* (also known as *OptoA1AR-EYFP*) was amplified from the pcDNA3.1/Optoα1AR-EYFP plasmid (Airan et al., 2009) (gift from Dr. Karl Deisseroth, Stanford University) and was subcloned into the FseI site of the Ai9 plasmid (Addgene). Then, the resultant *Opto*α*1AR-EYFP*-WPRE-bGH polyA fragment was amplified by PCR and was subcloned between the XhoI and EcoRV sites of the pCR-FRT-*Amp*-FRT plasmid (Yaguchi et al., 2014) (gift from Dr. Kunio Yaguchi). A bacterial artificial chromosome (BAC) clone, RPCI-23-361H22 (Regan et al., 2007) (BAC PAC Resources), containing the *GLT1* gene was modified by the Red/ET recombination system (Gene Bridges) to insert *Opto*α*1AR-YFP*-WPRE-polyA-FRT-*Amp*-FRT to the immediate downstream of the initiation codon. After colony selection by ampicillin, the *Amp* cassette was removed from the recombinant BAC clones by introducing the Flp recombinase expression plasmid 706-FLP (Gene Bridges). The final BAC construct was amplified, purified with the Large-Construction kit (Qiagen) and linearized by AscI digestion. Correct modification of the BAC was verified by pulsed-field gel analysis of restriction digestions and direct sequencing of the insert. The linearized BAC DNA was purified, adjusted to be ~1 ng/μl in a microinjection buffer (10 mM Tris-Cl, 0.1 mM EDTA, 100 mM NaCl, pH 7.4), and individually injected into the pronuclei of 445 C57BL/6J-fertilized embryos. As a result, 81 founders were born, of which 11 founders were positive for the transgene. Positive founder mice were identified by 301 bp DNA amplified by PCR using the following primer pair: 5' CGAGGCGCTAAAGGGCTTACC 3' and 5' CCCCAGCATAATCAGAAGGA 3'. Positive founder mice were crossed with C57BL/6J mice and maintained on this genetic background. The established 11 line gave astrocytic expression of Optoα1AR-EYFP with different expression strengths and variable positive cell proportion. Among them, the two lines, #941 and #877, were selected based on the selective expression of Optoα1AR-EYFP in astrocytes and used in the current study as strong TG and patchy TG mice, respectively. These heterozygous mice were crossed with C57BL/6J mice to obtain heterozygous mice for experiments and were noted as TG mice.

### Immunohistochemistry

Mice were transcardially perfused with 4% paraformaldehyde in 0.1 M phosphate buffer. Coronal sections of 60 μm thickness across AP ~−1.5 mm were obtained. These sections were incubated with anti-S100β (1:200, ab52642, Abcam), anti-NeuN (1:2000, MAB377, Chemicon-Millipore), anti-GFAP (1:2000, Z0334, DAKO) or anti-Iba1 (1:400, 019-19741, Wako) antibodies. For YFP signal enhancement in Supplementary Figure 8, polyclonal rabbit antibody (1:1000, Tomioka and Rockland, 2006) and monoclonal rat antibody (1:1000, Nacalai 04404-84) were used separately. Subsequently, sections were incubated with Alexa Fluor-594 conjugated secondary antibodies (1:1000, Molecular Probes-Invitrogen). Fluorescence images were obtained with a 10 × (0.4 NA) or 60 × (1.3 NA) objective lens using an Olympus FV-3000 confocal microscope.

### *In vivo* Two-Photon Ca^2+^ Imaging of Astrocytes

Adult Optoα1AR patchy TG mice (>2 months old) were anesthetized with 1.5 g/kg urethane. A metal frame was attached to the skull using a dental acrylic (Fuji LUTE BC, GC and Super Bond, Sun Medical), and a craniotomy (diameter 2.0–3.0 mm) for imaging was made above the somatosensory cortex (AP −1.5 to −2.5 mm and ML 1.5 to 2.5 mm). The dura mater was carefully removed, and the exposed cortex was loaded with Rhod-2 AM (0.4 mM, Molecular Probes-Invitrogen) for 1 h. After washing with HEPES-ACSF (125 mM NaCl, 3.5 mM KCl, 10 mM glucose, 10 mM HEPES, 2 mM CaCl_2_ and 2 mM MgSO_4_, pH 7.3) several times, the craniotomy was covered with agarose (2% w/v in HEPES-ACSF) and sealed by a thin glass coverslip. A Bergamo based two-photon microscope (Thorlabs) attached to a Chameleon Ultra two laser (Coherent) with 25 × objective lens (1.05 NA, Olympus) was used. The microscope is equipped with a reverse dichroic mirror (405/473-488/561/705-1600nm notched dichroic mirror, Thorlabs) and the emission light was separated by a dichroic mirror (FF562-Di03, Semrock), with band-pass filters FF03-525/50 and FF01-607/70 (both from Semrock) for the green and red channels, respectively. Rhod-2 fluorescence from 50 to100 μm below the pial surface was acquired using ThorImageLS software at a frame rate of 5 Hz. Rhod-2 or EYFP was imaged with 820 or 940 nm laser, respectively.

Photo-activation for Optoα1AR was carried out by 470 nm LED (M470L3, Thorlabs) after systemic 9-cis-Retinal supply. Strong (1 mW) or weak (0.1 mW) LED was targeted to an imaged region (φ = ~0.8 mm) through the objective lens. To protect photomultiplier tubes (PMTs) from LED illumination, the optical path to the PMTs was blocked by a built-in mechanized shutter. 9-cis-Retinal (R5754, Sigma) was dissolved in Dimethyl sulfoxide (DMSO) to make 200 mM solution and stored frozen in 5 μL aliquots. On the day of experiment, an aliquot (i.e., containing 0.28 mg 9-cis-Retinal) was diluted in 100 μL HEPES-ACSF or saline at 35°C and administered by intraperitoneal (i.p.) injection. This amount of 9-cis-Retinal is comparable to that in the previous study, where i.p. injection of 0.375 mg of 9-cis-Retinal restored electroretinogram responses in endogenous 11-cis-retinal deficient mice (Parker et al., 2009). LED-induced Rhod-2 responses were reliably observed ~30 min after 9-cis-Retinal injection and thereafter for ~1.5 h. LED illuminations were repeated at ~9 min interval. Tail pinch was manually applied *via* blunt tongs for ~1 s.

For long-term Ca^2+^ imaging, RCaMP1.07 was selectively expressed in astrocytes under the control of a GFAP promoter. Optoα1AR strong TG mice (>2 months old) were anesthetized with ketamine and xylazine (56 and 8 mg/kg, respectively) and a metal frame was attached to the skull. A small craniotomy was made above the somatosensory cortex and a glass micropipette containing AAV9-hGFAP-RCaMP1.07 (3.0–4.0 × 10^12^ vg/ml, PBS) was inserted to a depth of 300 μm below the pial surface. Microinjection of 300 nl was made over 3 min using a Femtojet injector (~5 psi, Eppendorf), and the exposed cortical surface was covered by a sterilized round cover glass (3 or 4 mm in diameter) to be used as a cranial window for later imaging.

>2 weeks later, AAV-microinjected mice were anesthetized with 1.5 g/kg urethane, and RCaMP imaging was performed using the Bergamo two-photon microscope with 1,040–60 nm laser. In these experiments, the same region was repeatedly imaged before and after LED illuminations (1 mW) with different durations (1 s, 3 s, or 3 min) at 9 min interval. For 3 min illumination, 1.5 s LED-on and 0.5 s LED-off, were repeated. RCaMP signals were first imaged without 9-cis-Retinal addition. Thereafter, 9-cis-Retinal was supplemented by i.p. injection, and imaging was resumed after 40 min. For testing the repeatability of 1-s Optoα1AR activation, LED illuminations were repeated at 3-min intervals in addition to 9-min interval.

### *In vivo* Two-Photon Ca^2+^ Imaging of Neurons

AAV1.Syn.NES-jRGECO1a.WPRE.SV40 (Penn Vector Core, 3.4–5.7 × 10^12^ vg/ml) was injected at a depth of 300 μm of the somatosensory cortex of adult WT and Optoα1AR strong TG mice, as above. Following >2 weeks recovery, mice were imaged using the Bergamo microscope with 1040–60 nm laser at a frame rate of 5 Hz in anesthetized (1.5 g/kg urethane) or awake condition. In the latter case, mice were trained to be restrained under the microscope using a mechanical fixture that rigidly fixes the head frame once a day for 5–7 days. During this training, mice were deprived of water, and had access to water once the head frame is fixed to the apparatus, thereby making an association between the head fixture and satiation of thirst. In both anesthetized and awake conditions, 9-cis-Retinal i.p. injection was made ~40 min before imaging. Optoα1AR was activated by LED (1 mW, 1 s), which was repeated four to eight times at 9-min interval. In some TG mice, adenosine A1R antagonist, CPT (C102, Sigma) was administered (20 mg/kg, i.p., dissolved at 40 mM in DMSO) 60 min before optogenetic stimulation.

### *In vivo* Local Field Potential Recording

Adult WT and Optoα1AR strong TG mice (>2 months old) were anesthetized with isoflurane (1.5%). A metal frame was attached to the skull and mice were rigidly fixed in a headplate holding device. A craniotomy was made above the somatosensory cortex. A glass micropipette (2 μm tip diameter, 1B150F-4, World Precision Instruments) filled with HEPES-ACSF was placed to an electrode holder with a headstage preamplifier. The headstage is then mounted to a remote-controlled micromanipulator (EMM-3NV, Narishige). Under a stereo microscope, the glass micropipette was inserted to the primary somatosensory cortex, trunk region (AP ~−1.5 mm, ML ~1.7 mm and DV ~0.25 mm) (Paxinos and Franklin, 2001). An optical fiber (200 μm diameter, CFML22L10, Thorlabs) connected to 470 nm LED devices (LEDFRJ-B_FC and LEDRV_1CH_1000, Doric) was placed over the pial surface above the recording site. Two vitrodes (L150, Nihon Kohden) were put on the both sides of the dorsal trunk to apply sensory stimulation (1.5 mA, duration 1 ms, interval 10 s). After 9-cis-Retinal i.p. injection, isoflurane dosage was decreased to ~0.8 %. Thereafter, the room light was turned off.

After sensory evoked response was stabilized (typically ~1 h), evoked field potential recording began (Multiclamp 700B, Axon instruments; 1,000×, 0.1 Hz to 3 kHz, digitized at 10 kHz using a LabVIEW-based data acquisition system, National Instruments). After stable evoked field potential response was obtained, brief LED illuminations were delivered above the recording site (1mW, duration 1 s, interval 5 min, six times).

### Behavioral Experiments

Adult Optoα1AR TG mice and littermate WT mice (>2 months old) were anesthetized with ketamine and xylazine (56 and 8 mg/kg, respectively), and wireless LED device containing two LEDs (φ3.1, ~8.5 mW ×2) (Iwai et al., 2011) was secured to skull above the anterior cortex (AP ~1.5 mm, ML ~1.5 mm) with dental cement. After >2-week recovery, mice were habituated to attachment of the LED-receiver and -battery with 9-cis-Retinal or vehicle i.p. injection for ~1-week (duration ~1 h, interval 2-3 day, 4 times). This LED device attachment and systemic injection of retinal or vehicle were always done 40 to 60 min before the following behavioral tests. LED illumination began ~1 min after mice were put in the behavioral chambers.

#### Novel Open Field Test

Individual mice were placed in the center of a novel open field (40 × 40 cm) and allowed to freely explore the arena for 45 min. The anterior cortex was transcranially illuminated by the LED device (duration 3 s, interval 3 min, 15 times). In some TG mice, adenosine A1R antagonist, DPCPX (C101, Sigma) dissolved at 4 mg/mL in DMSO was i.p.-injected at the dosage of 1 mg/kg, 40 to 90 min before LED illuminations.

#### Y-Maze Test

Individual mice were placed in the center of a Y-maze (YM-40M, BrainScience idea) and allowed to freely explore the maze for 15 min with the LED illuminations (duration 3 s, interval 3 min, five times) delivered.

#### Novel Object Recognition Test

This test consisted of three different phases: habituation, training and test. Individual mice were first habituated to an open field with a plywood floor (39.5 × 39.5 cm), with 10 min of exploration three times in three consecutive days. The plywood floor was changed for each mouse, and the plywood floor was consistently used for each mouse in later training and test. On the training day, individual mice were placed in the habituated open field for 10 min, where two identical objects were fixed 20 cm apart on the plywood with double-sided adhesive tape (SRE-19, 3M). Three different objects of similar size were used in a counter-balanced manner, as the role (familiar vs. novel object) as well as the position (left or right) were randomly permuted. Transient LED illuminations (duration 3 s, interval 3 min, four times) or longer LED illuminations (duration 30 s, interval 3 min, four times) were delivered during the training period. Immediately after training, mice were returned to the home cage, and LED-receiver and -battery were detached from mice 10 min later. In post-activation experiments, LED illuminations (duration 3 s, interval 3 min, four times) were delivered during this 10 min in the home cage immediately after training. On the test day (1 day or 14 days after training), mice were exposed to one of the pre-familiarized objects and a novel object for 10 min without LED illumination. In some TG mice, DPCPX was i.p.-injected at the dosage of 1 mg/kg, ~60 min before LED illuminations.

#### Conditioned Place Preference Test

The conditioned place preference apparatus consisted of three chambers. The left and right chambers have the same size (25 × 25 cm) but were distinguished by their walls with vertical-stripes or horizontal-stripes, respectively. The center chamber (25 × 15 cm) has neutral walls without stripe. This test consisted of three different phases: pre-test, conditioning and post-test. On day 1 (for pre-test), individual mice were placed in the center chamber and allowed to freely enter and explore the three chambers for 20 min without LED illumination to measure default place preference. On day 2 to 5 (for conditioning), mice pre-treated with 9-cis-Retinal were confined for 30 min to either the left or right chamber on alternate days, and LED illuminations (duration 3 s, interval 3 min, 10 times) were delivered in either the left or right chamber. Days with LED illuminations (day 2 and day 4 or day 3 and day 5) were counterbalanced. On day 6 (for post-test), mice were again placed in the center chamber and allowed to freely enter and explore the three chambers for 20 min without LED illumination to measure the post-conditioning place preference. Post-test and pre-test were conducted identically.

### Data Analysis

#### Immunohistochemistry

Quantifications (Supplementary Figure 1) were performed from layer 1 to 6 in both sides of 500 μm-wide somatosensory cortex (ML ~1.5–2.0 mm). Among the cellular marker-positive cells, EYFP-positive or EYFP-negative cells were manually counted from 60 × images at single plane. Intensity of GFAP, Iba1, or EYFP of the above somatosensory cortical area was measured by ImageJ software from stack images acquired with a 10 × objective over 60 μm-thick brain slices. Measured intensity of GFAP or Iba1 of a mouse was normalized by mean intensity of WT mice and presented as relative intensity in Supplementary Figures 1C,D. Measured intensity of EYFP of a TG mouse was first subtracted by mean intensity of WT mice which corresponds to background EYFP intensity. The subtracted value was then divided by the mean intensity of patchy TG mice and described as relative percent to patchy TG mice in the text.

#### Two-Photon Imaging

Analysis was performed by ImageJ and MATLAB software. Image shift in xy axis was adjusted by the TurboReg ImageJ plug-in program for all images.

**Figure 1:**
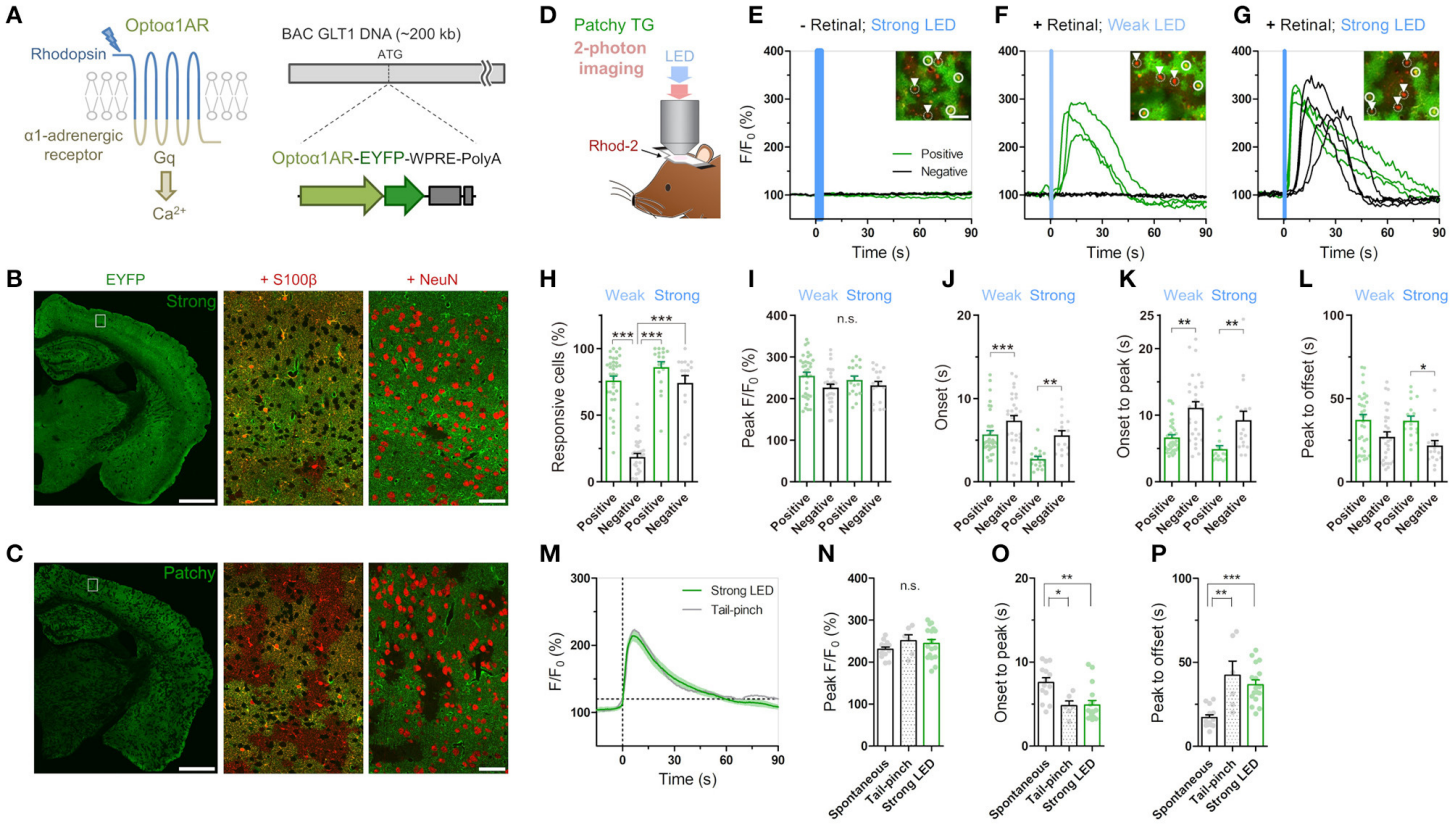
Cis-Retinal supplement is required for reliable Optoα1AR activation by brief illumination *in vivo*. **(A)** Optoα1AR, optically-activatable Gq-GPCR, is a chimeric molecule of mammalian rhodopsin and Gq-coupled α1 adrenergic receptor (Airan et al., 2009), which induces intracellular Ca^2+^ elevation upon activation. For astrocyte-selective expression, a TG vector was constructed with the BAC GLT1 DNA (Regan et al., 2007). **(B)** Line #941 (“Strong”) shows intense EYFP fluorescence (green) throughout the brain. High magnification view in white rectangle shows that EYFP is expressed in S100β-positive astrocytes (red). Relatively high EYFP signals are visible in astrocytic somata and endfeet. By contrast, hardly any NeuN signals (red) from neurons overlap with EYFP signals. Scale bar: 1 mm (left), 50 μm (middle and right). **(C)** Line #877 (“Patchy”) shows visible EYFP fluorescence (green) in a patchy pattern throughout the cortex, hippocampus and striatum. High magnification view in white rectangle shows that EYFP signals colocalize with S100β signals (red) in roughly half of the astrocytes. There are EYFP-negative but S100β-positive domains. Relatively high EYFP signals are visible in astrocytic somata and endfeet. NeuN signals (red) do not overlap with EYFP signals. Scale bar: 1 mm (left), 50 μm (middle and right). **(D)** Sketch of *in vivo* astrocytic Ca^2+^ imaging with LED illumination in cortical superficial layers of urethane-anesthetized patchy-TG mice. Astrocytes are loaded with the red Ca^2+^ indicator Rhod-2. **(E–G)** Example plots of *in vivo* astrocytic Ca^2+^ imaging with optical stimulation. Optoα1AR-positive and negative astrocytes (white circles and arrowheads, respectively) are analyzed based on the EYFP expression. Green and black traces correspond to EYFP-positive and -negative astrocytes, respectively. Insets: images of cells analyzed in the respective plots. Scale bars: 50 μm. **(E)** Astrocytic Ca^2+^ imaging without retinal addition. Strong blue LED illumination (1 mW, 5 s) did not induce Ca^2+^ elevations in all the encircled six astrocytes. **(F)** Astrocytic Ca^2^ imaging with retinal. Weak LED illumination (0.1 mW, 1 s) induced a transient Ca^2+^ increase in EYFP-positive astrocytes, but not in EYFP-negative astrocytes. **(G)** Astrocytic Ca^2^ imaging with retinal. Strong LED illumination (1 mW, 1 s) induced a rapid Ca^2+^ increase in EYFP-positive astrocytes. Delayed Ca^2+^ elevation was observed in EYFP-negative astrocytes. **(H–L)** Analysis of Ca^2+^ response in EYFP-positive and -negative astrocytes upon weak or strong LED illumination with retinal addition. Each symbol represents an individual imaging session (Weak LED: 35 sessions, nine patchy TG mice; Strong LED: 17 sessions, nine patchy TG mice). **(H)** Proportion of responsive cells. The weak-negative group was the least responsive (***p < 0.001, Tukey's test after one-way ANOVA). **(I)** Peak amplitude was similar for all groups (*p* > 0.11, one-way ANOVA). **(J,K)** Onset time and onset-to-peak time were shorter in the positive group for both stimulation strengths (***p < 0.001, **p < 0.01, Dunn's test after Kruskal-Wallis one-way ANOVA). **(L)** Peak-to-offset time was shorter in the strong-negative group (*p < 0.05, Dunn's test after Kruskal-Wallis one-way ANOVA). **(M–P)** Comparison of spontaneous, tail-pinch-induced, and optogenetically induced (strong illumination) Ca^2+^ response. **(M)** Mean and SEM trace of optogenetically induced (green) and tail-pinch-induced (gray) Ca^2+^ increase. Time 0 corresponds to onset time, when F/F_0_ reaches 120%. **(N)** Peak amplitude was similar among the 3 groups (*p* > 0.32, one-way ANOVA). **(O,P)** Onset-to-peak and peak-to-offset times of Ca^2+^ events were similar between the tail pinch and optogenetically induced groups (*p* > 0.05, Tukey's test after one-way ANOVA) and distinct from spontaneously observed Ca^2+^ events (***p < 0.001, **p < 0.01, *p < 0.05, Tukey's test after one-way ANOVA). Each symbol represents an individual imaging session (Spontaneous: 14 sessions, 10 TG mice; Tail-pinch: six sessions, four TG mice).

Rhod-2 and RCaMP signals in astrocytes (Figure 1; Supplementary Figure 2) were extracted from cell bodies manually on ImageJ, and these intensity data in region of interests (ROIs) were exported to MATLAB software for further analysis of F/F_0_, where F is fluorescence intensity within a given ROI at each time point and F_0_ is the mean fluorescence intensity within a given ROI during 0-1 min before LED illumination. A responsive cell was defined as a cell exhibiting >120% F/F_0_ for >10 s within 30 s after LED illumination. Peak F/F_0_, onset time, peak time and offset time were analyzed for responsive cells. Onset time is the time firstly reaching 120% F/F_0_. Offset time is a time firstly returning to 120% F/F_0_ after peak. The values in each image were averaged across ROIs, and these normalized values were presented in Figure 1; Supplementary Figure 2.

**Figure 2:**
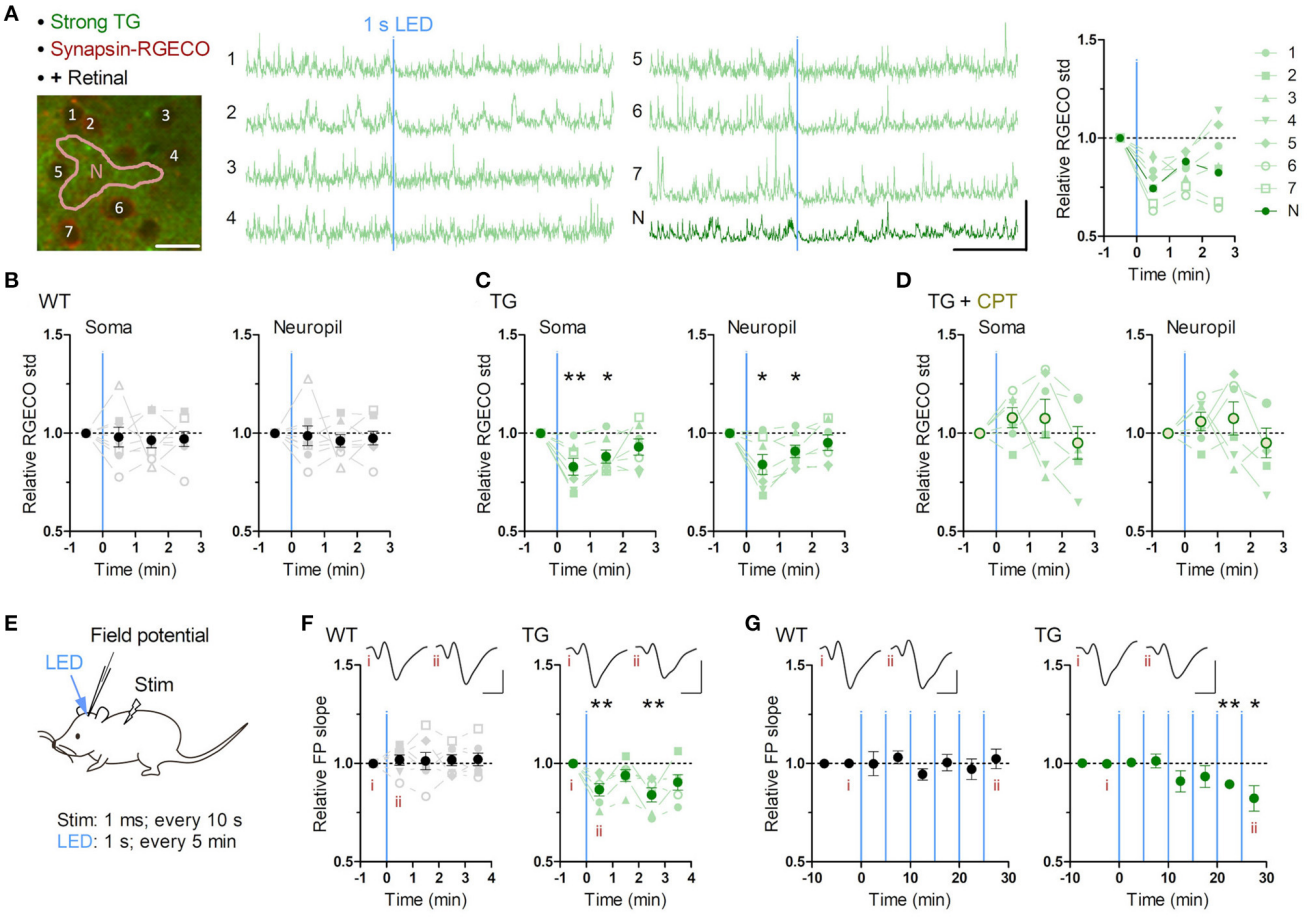
Brief astrocytic Gq activation suppresses neuronal activity. **(A–D)** Neuronal Ca^2+^ imaging from somatosensory cortex layer 2/3 in awake mice with optogenetic induction of Gq signaling in astrocytes. **(A)** Representative two-photon image of somatosensory cortex of a strong TG mouse expressing jRGECO1a (RGECO) in neurons by AAV-Syn-jRGECO1a (left). RGECO F/F_0_ from the labeled somata (1-6) and neuropil (N) decreased rapidly after 1 s LED illumination (middle). Ca^2+^ activity, measured as the standard deviation (std) of RGECO F/F_0_, decreased in the first and the second 1 min after LED illumination (right). Scale bars: 20 μm (micrograph); 100% F/F_0_ and 1 min (traces). **(B)** Ca^2+^ activity of neuronal somata and neuropil in WT mice did not change after LED illumination (*p* > 0.70 and *p* > 0.80, paired *t*-test, 1 min after LED illumination vs. 1 min before LED illumination, eight mice). **(C)** Ca^2+^ activity of neuronal somata and neuropil in TG mice decreased in the first and second minutes after LED illumination (first minute: *p* < 0.008 and *p* < 0.03; second minute: *p* < 0.02 and *p* < 0.03, paired *t*-test vs. 1 min before LED illumination, seven mice). **(D)** Adenosine A1R antagonist CPT blocked Optoα1AR-induced neuronal Ca^2+^ activity decrease in somata and neuropil (*p* > 0.18 and *p* > 0.25, paired *t*-test, 1 min after LED illumination vs. 1 min before LED illumination, six mice). **(E–G)** Sensory evoked field potential (FP) recording in somatosensory cortex layer 2/3 of shallowly anesthetized mice upon LED illumination. **(E)** FP response was evoked by sensory stimulation to the trunk (duration 1 ms, interval 10 s) before and after brief LED illumination (1 mW, duration 1 s). Six optical stimulations (5 min interval) were performed in a session. **(F)** LED time-triggered averaging of FP slope shows a reduction of sensory evoked response after astrocytic Gq activation in the first 1 min (*p* < 0.008, paired *t*-test vs. 1 min before LED illumination, six TG mice). WT mice did not show a significant change in FP slope (*p* > 0.51, paired *t*-test vs. 1 min before LED illumination, seven WT mice). This reduction in TG mice was detectable 3 min after LED illumination (*p* < 0.008, paired *t*-test vs. 1 min before LED illumination, six TG mice). Insets: averaged FP traces from a representative mouse, with the left and right traces averaged within 1 min before and 1 min after LED illumination, respectively. Scale-bars: 200 μV and 20 ms. **(G)** In the 30 min recording, evoked FP slope gradually decreased in TG mice (20–25 min and 25–30 min periods: *p* < 0.004 and <0.05, paired *t*-test vs. 0–10 min before LED illumination, six TG mice), while that in WT mice did not change throughout the 30 min period (*p* > 0.1, paired *t*-test vs. 0–10 min before LED illumination, seven WT mice). Insets: averaged FP traces from a representative mouse, with the left and right trace averaged within the 5 min period before the first LED illumination and the 5 min period after the last LED illumination, respectively. Scale bars: 200 μV and 20 ms. *p < 0.05, **p < 0.01.

RGECO signals in neurons (Figure 2; Supplementary Figure 3) were extracted from cell bodies and neuropils manually on ImageJ, and these intensity data were analyzed by MATLAB to calculate F/F_0_ and standard deviation (std) of F/F_0_ in each ROI. These stds were averaged across ROIs and then further averaged across imaging trials. The mean std in each mouse was normalized by that in 0-1 min before LED illumination as 1, and this normalized value was presented as relative RGECO std in Figure 2; Supplementary Figure 3.

#### Local Field Potential Recording

The slope of evoked LFP (Figure 2) was calculated by MATLAB as described previously (Takata et al., 2011; Monai et al., 2016). First, the initial deflection of the LFP response was isolated. Next, the region for slope calculation was defined as the interval within 20 to 80% of the peak-to-peak amplitude of the negative deflection. The slope was computed by linear regression of the selected region. LFP slope within 1 min bin in each mouse was averaged with regard to 6 times LEDs, and this mean value was normalized by that in 0-1 min before LED illumination as one, and this normalized value was presented as relative FP slope in Figure 2F. Averaged LFP slope within 5 min bin in each mouse was normalized by that in 0-5 min before LED illumination as one, and this normalized value was presented as relative FP slope in Figure 2F.

#### Behavioral Experiments

**Figure 3:**
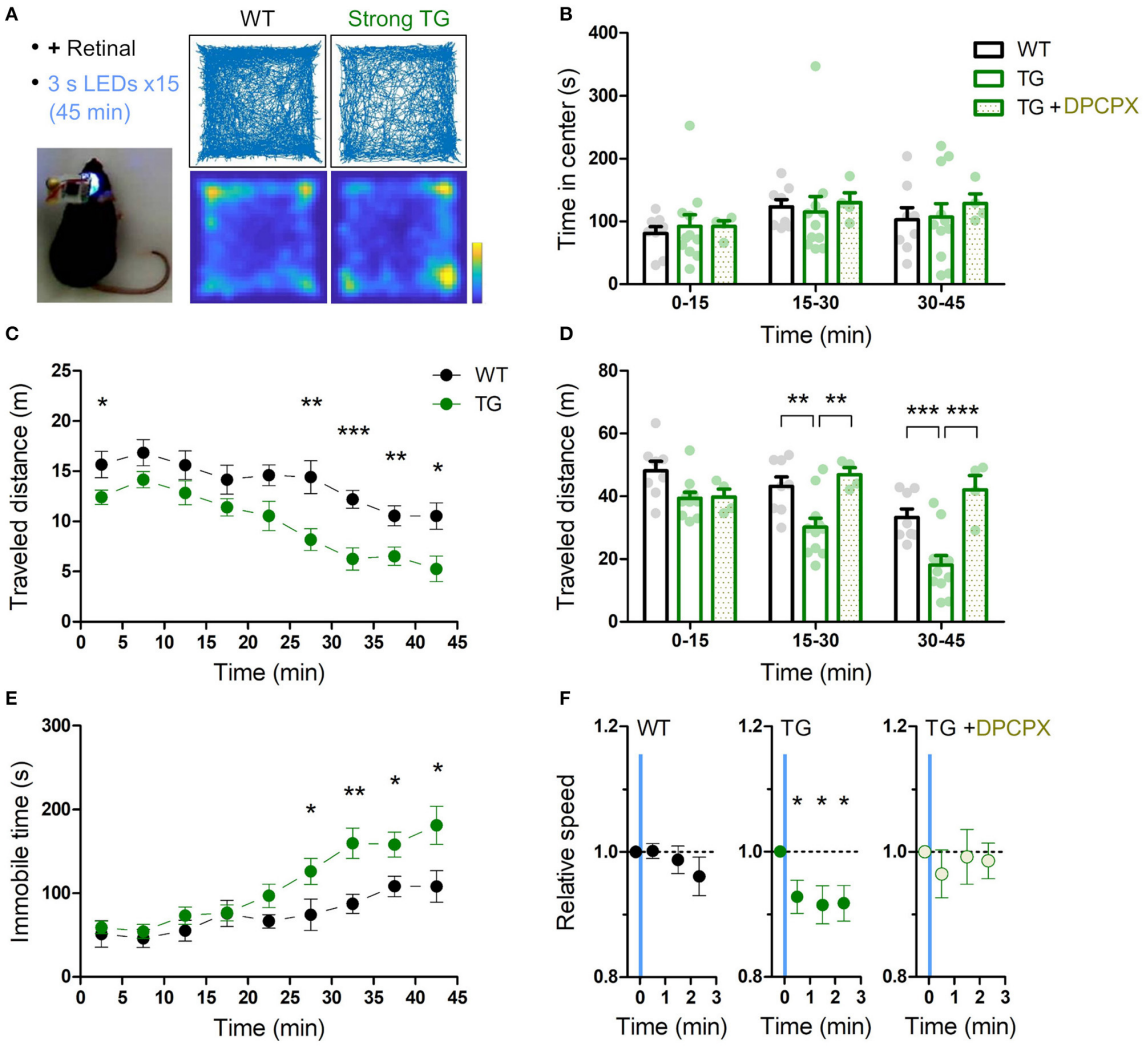
Transient astrocytic Gq activation in the anterior cortex decreases locomotion in a novel open-field. **(A)** Anterior cortical areas of freely behaving mice were illuminated by a wireless LED device. Representative trajectories and occupancy maps during 45 min open-field behavior of a WT or a strong TG mouse. Both mice received LED illuminations (duration 3 s, interval 3 min, 15 times) with retinal pre-treatment (i.p.). The TG mouse traveled a shorter distance, while spending a similar length of time in the center zone as the WT mouse. Color bar: 15 s. **(B)** Time in the center zone was not significantly different between experimental conditions and between 15 min periods (*p* > 0.75 and *p* > 0.17, two-way ANOVA, 8 WT mice, 11 strong TG mice vs. four strong TG mice with DPCPX). **(C)** TG mice gradually exhibited shorter traveled distances (***p < 0.001, **p < 0.01, *p < 0.05, unpaired *t*-test, 8 WT mice vs. 11 strong TG mice). **(D)** Traveled distance in TG mice was significantly shorter in 15–30 min and 30–45 min, which was reinstated by DPCPX injection (**p < 0.01, ***p < 0.001, Bonferroni test after two-way ANOVA). **(E)** TG mice gradually increased immobile time (*p < 0.05, **p < 0.01, unpaired *t*-test, 8 WT mice vs. 11 strong TG mice). **(F)** LED-triggered averaging indicates a rapid and lasting decrease of locomotion in TG mice. Locomotion speed of TG mice was significantly reduced in 0–60 s, 60–120 s, and 120–160 s after LED illumination in comparison with that in 0-20 s before LED illumination (*p* < 0.03, *p* < 0.02, and *p* < 0.02, paired *t*-test). Locomotion speed of WT mice or TG mice with DPCPX did not change significantly after LED illumination (*p* > 0.2 or *p* > 0.4, paired *t*-test).

**Figure 4:**
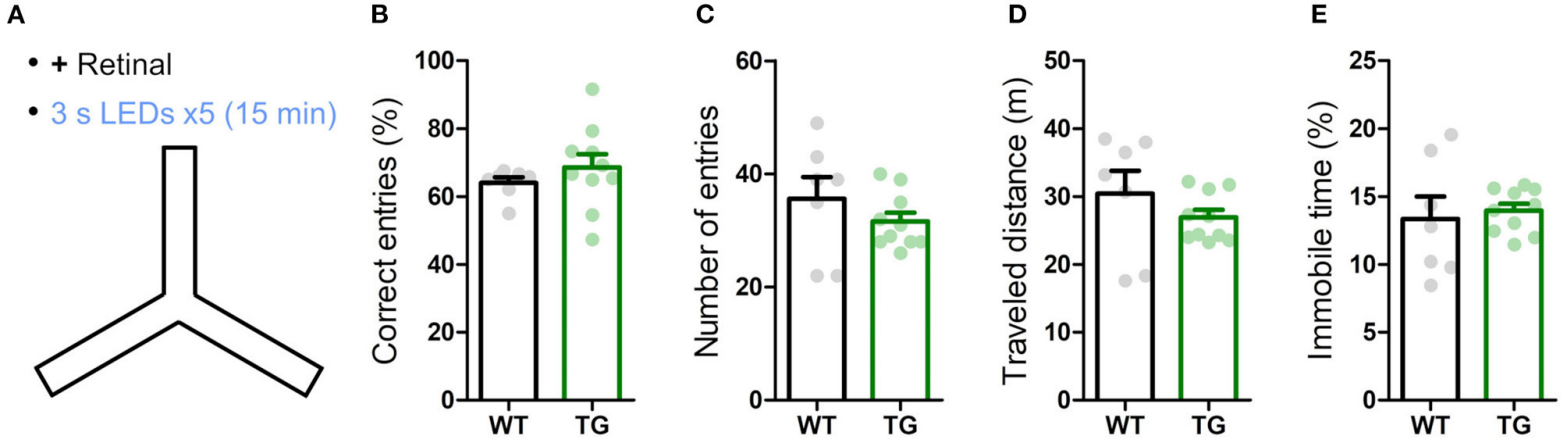
Transient astrocytic Gq activation in the anterior cortex does not affect short-term memory in a Y-maze. **(A)** WT and strong TG mice were pre-treated with retinal and were put in a Y-maze for 15 min with transient LED illuminations (duration 3 s, interval 3 min, five times) delivered. **(B)** Percentage of correct arm entries (unique triplets) was not significantly different between WT and TG mice (*p* > 0.30, Welch's *t*-test, 7 WT mice vs. 10 strong TG mice), but the variance was higher in TG mice (*p* < 0.02, *F*-test). **(C–E)** Number of arm entries, traveled distance and immobile time did not differ between WT and TG mice (*p* > 0.37, *p* > 0.35, and *p* > 0.73, Welch's *t*-test; *p* < 0.05, *p* < 0.02, and *p* < 0.02, *F*-test).

**Figure 5:**
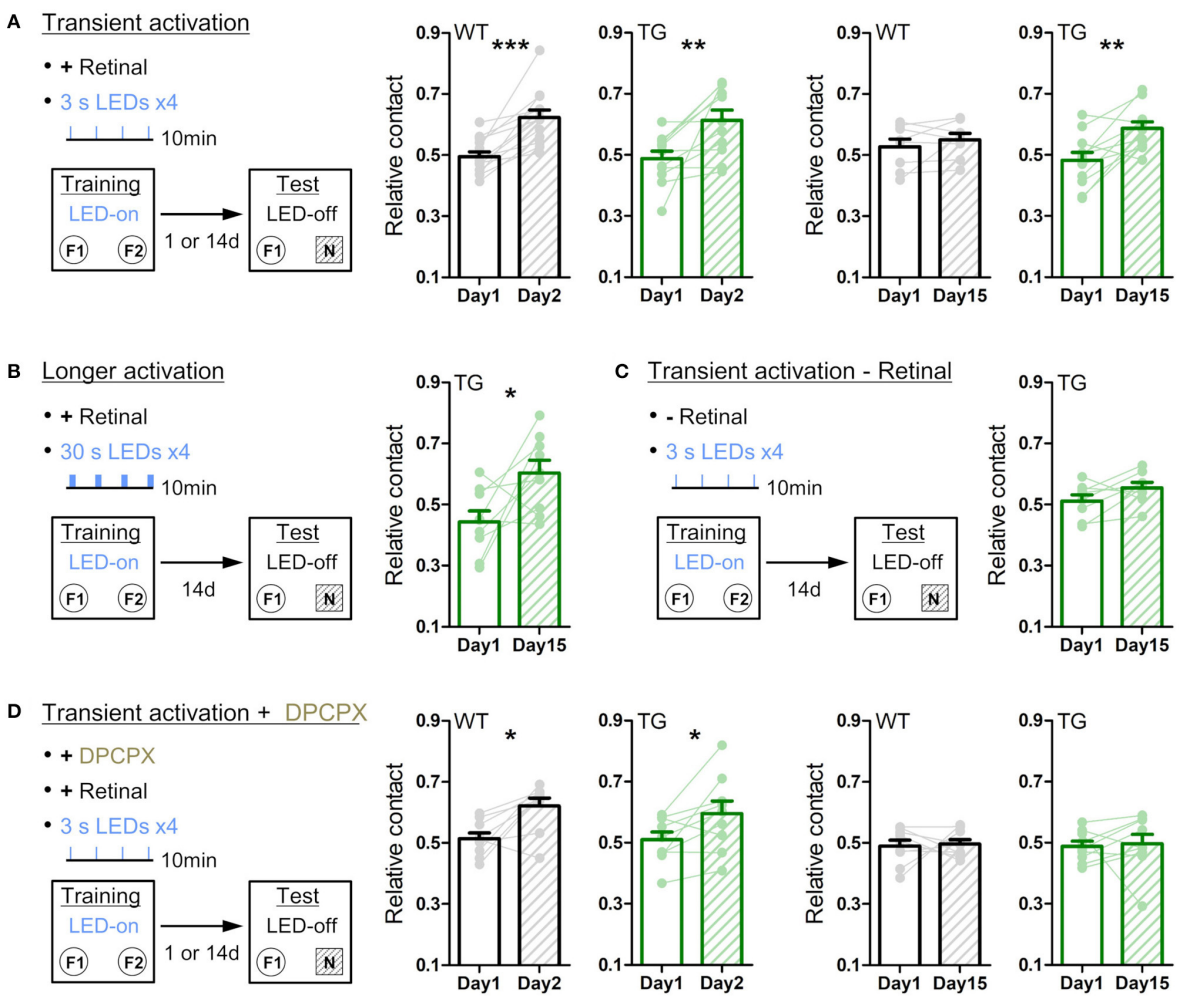
Transient astrocytic Gq activation in the anterior cortex enhances long-term object recognition memory. **(A)** Transient activation protocol. On the training day, WT or strong TG mice were placed in an open-chamber for 10 min, where two identical objects (F1 and F2) were placed apart. Mice were pre-treated with retinal. Transient LED illuminations (duration 3 s, interval 3 min, four times) were delivered during the training period. On the test day (1 day or 14 days after training), mice were exposed to one of the pre-familiarized objects (F1) and a novel object (N) for 10 min without LED illumination. In the 1-day test, both WT (black) and TG (green) mice similarly increased relative contact time to the novel object (*p* < 0.0002 and *p* < 0.08 paired *t*-test vs. relative contact time to F2 object in the training, 13 WT mice and 11 TG strong mice). In the 14-day test, TG mice still retained the novel object preference (*p* < 0.005, paired *t*-test, 11 strong TG mice), whereas WT mice did not (*p* > 0.31, paired *t*-test, eight WT mice). **(B)** Longer activation protocol. The training procedure is the same as in A, except for longer LED illuminations (30 s) delivered. In the 14-day test, TG mice showed the novel object preference (*p* < 0.04, paired *t*-test, nine strong TG mice). **(C)** Transient activation without retinal pre-treatment. The training procedure is the same as in A, except for pre-injection of vehicle instead of retinal. In the 14-day test, TG mice did not show the significant novel object preference (*p* > 0.1, paired *t*-test, eight strong TG mice). **(D)** Transient activation with DPCPX protocol. The training procedure is the same as in A, except for additional adenosine A1R antagonist DPCPX pre-treatment. In the presence of DPCPX, the transient LED illuminations did not induce the novel object preference 14 days later (*p* > 0.82 and *p* > 0.82, paired *t*-test, nine WT mice and nine strong TG mice), whereas novel object preference was expressed 1 day after familiarization (*p* < 0.02 and *p* < 0.04, paired *t*-test, 9 WT mice and nine strong TG mice). *p < 0.05, **p < 0.01, ***p < 0.001.

The entire sessions in behavioral experiments were recorded by a video camera (C910, Logicool). Animal's body position was determined by Any-maze behavior tracking software (Stoelting). Regarding novel open field test (Figure 3; Supplementary Figures 4, 5), time in the center zone (central 20 x 20 cm), total traveled distance and immobile time were calculated by Any-maze software. Speed of traveled distance in each mouse was averaged with regard to 15 times LEDs, and this mean value was normalized by that in 0–20 s before LED illumination as one, and this normalized value was presented as relative speed in Figure 3F. Regarding Y-maze test (Figure 4), total number of arm entries and correct arm entry (the entry to the arm different from the current and immediate prior ones) were determined from the recorded video. Total traveled distance and immobile time were measured by Any-maze software. Regarding novel object recognition test (Figure 5; Supplementary Figure 6), contact time was defined as time in touching the objects with nose or forepaws, which was determined from the recorded video. Relative contact is a ratio of contact time to a replaced object (F2 or N in Figure 5) to total contact time to both objects. Traveled distance was measured by Any-maze software. Regarding conditioned place preference (Supplementary Figure 7), times in the individual chambers were measured by Any-maze software. Relative time in LED chamber is a ratio of time in LED-illuminated chamber (left chamber in Supplementary Figure 7) to time in both left and right chamber.

### Statistics

Statistical analyses were performed by Prism and MATLAB software. Comparisons between two groups were analyzed by unpaired *t*-test. If variances were significantly different between them, Welch's correction was applied (Welch's *t*-test). For comparisons of data between before and after a manipulation, paired *t*-test was used. Comparisons of three or more groups were performed by one-way ANOVA with *post-hoc* Tukey's test. If variances were significantly different between them, Kruskal-Wallis one-way ANOVA with *post-hoc* Dunn's test was applied. For two-factor comparisons, two-way ANOVA with *post-hoc* Bonferroni test was used. All values are expressed as mean ± SEM.

## Results

### Transgenic Mice With Selective Astrocytic Expression of Optoα1AR

We have generated TG mice in which Optoα1AR (Airan et al., 2009) is expressed under the control of a BAC-GLT1 promoter (Regan et al., 2007) (Figure 1A). Among the 11 GLT1-Optoα1AR-EYFP founders, a few lines showed astrocyte-specific expression. We examined two lines, #941 and #877, in this study. Line #941 showed ubiquitous expression of Optoα1AR-EYFP in astrocytes throughout the brain (Figure 1B; Supplementary Figure 1A), whereas line #877 had expression in a sparse population of astrocytes (Figure 1C; Supplementary Figure 1B). EYFP fluorescence in the cortex of line #941 was twice as strong as that of line #877 (195.3 ± 9.0% of 9 strong TG mice relative to 100 ± 7.8% of 10 patchy TG mice; *p* < 0.0001, unpaired *t-*test). Accordingly, #941 and #877 are referred to as “strong” and “patchy” TG mice, respectively. Immunohistochemistry with astrocytic (S100β), neuronal (NeuN) and microglial (Iba1) markers showed that Optoα1AR-EYFP was expressed in astrocytes (strong TG: 76.9 ± 3.3%, S100β-positive 448 cells, four mice; patchy TG: 49.1 ± 2.8%, 476 cells, 4 mice). EYFP expression was negligible in neurons (strong TG: 8.3 ± 1.7%, NeuN-positive 2,463 cells, four mice; patchy TG: 1.4 ± 0.6%, 2,177 cells, four mice) and microglia (strong TG: 1.4 ± 0.8%, Iba1-positive 142 cells, four mice; patchy TG: 1.6 ± 0.9%, 151 cells, four mice) in the cortex (Supplementary Figures 1A,B). Cortical GFAP and Iba1 expressions were generally low in wild type (WT), strong and patchy TG mice (Supplementary Figure 1C, GFAP, strong TG: 89.5 ± 3.9%, patchy TG: 87.5 ± 2.4%, relative to WT: 100 ± 6.2%. Supplementary Figure 1D, Iba1, strong TG: 89.2 ± 4.5%, patchy TG: 87.1 ± 3.6%, relative to WT: 100 ± 5.7%, *n* = 4 mice for all groups). Fine ramified microglial processes were prevalently observed in Iba1 immunohistochemistry (Supplementary Figures 1A,B), suggesting that the expression of the foreign protein does not cause glial inflammation in these TG lines.

Next, we performed *in vivo* Ca^2+^ imaging from superficial layers of the cortex in urethane-anesthetized patchy TG mice. Cortical astrocytes were loaded with the red Ca^2+^ indicator Rhod-2 and observed by two-photon microscopy (Figure 1D). Optoα1AR-positive astrocytes were distinguishable by their EYFP fluorescence, allowing simultaneous investigation of Optoα1AR-positive and -negative astrocytes (Figures 1E–G, insets). Unlike channelrhodopsin-2, activation of the vertebrate rhodopsin-based GPCRs including the Optoα1AR requires cis-retinal, which is converted to trans-retinal and released upon activation (Travis et al., 2007; Redmond, 2009). Consistent with low levels of endogenous cis-retinal in the cortex, blue light illumination did not induce Ca^2+^ elevations in Optoα1AR-positive astrocytes even when power and duration of the blue light were increased (Figure 1E). Next, we repeated the experiment with a supplement of 9-cis-retinal by i.p. injection. Notably, systemic 9-cis-retinal administration enabled reliable photoactivation of Optoα1AR (Figures 1F,G; Supplementary Videos 1, 2). Upon brief blue light illumination (1 s, 0.1 mW) at the surface of the cortex through the objective lens, Optoα1AR-positive astrocytes elevated their Ca^2+^ levels with a delay of 5.71 ± 0.45 s from the onset of illumination (1,717 out of 2,162 cells, 35 sessions, nine mice; Figures 1F,H,J). On the other hand, a significantly lower proportion of simultaneously imaged Optoα1AR-negative astrocytes responded to the photostimulation (270 out of 1,484 cells, 35 sessions, nine mice; Figures 1F,H).

Remarkably, more intense illumination (1 s, 1 mW) gave rise to Ca^2+^ elevations in Optoα1AR-negative astrocytes (274/358 cells, 17 sessions, nine mice; Figure 1G; Supplementary Video 2). Comparison of Optoα1AR-positive and -negative astrocytes revealed that Optoα1AR-positive astrocytes had a similar amplitude (Figure 1I), a faster onset (2.76 ± 0.30 s vs. 5.59 ± 0.54 s; 646 out of 764 cells vs. 274 out of 358 cells, 17 sessions, eight mice; Figure 1J), and rise time (onset to peak: 4.92 ± 0.51 s vs. 9.28 ± 1.29 s; Figure 1K). The decay time was also longer in positive astrocytes (peak to offset: 36.7 ± 2.76 s vs. 21.8 ± 2.90 s; Figure 1L).

We compared optically evoked astrocytic Ca^2+^ elevations with spontaneous Ca^2+^ elevations which occur with low frequencies under urethane anesthesia (Hirase et al., 2004; Thrane et al., 2012). We found that although both magnitudes are similar, optically induced Ca^2+^ elevations are faster in the rise time and slower in the decay time than spontaneous Ca^2+^ events (optically evoked vs. spontaneous, peak F/F_0_: 244.8 ± 9.33% vs. 230.9 ± 5.17%; onset to peak: 4.92 ± 0.51 s vs. 7.58 ± 0.56 s; peak to offset: 36.7 ± 2.76 s vs. 17.2 ± 1.61 s; Figures 1N–P). On the other hand, optically induced Ca^2+^ elevations are similar to tail-pinch induced Ca^2+^ events (peak F/F_0_: 251.4 ± 13.7%; onset-peak: 4.85 ± 0.536 s; peak-offset: 42.3 ± 8.47 s; Figures 1M–P), suggesting that the optically-evoked Gq signaling mimics the salient stimuli-evoked *in vivo* event (Figure 1M).

We further investigated the optimal light duration for astrocytic Gq activation. To reliably image astrocytic Ca^2+^ level for a long period, RCaMP was expressed in layer 2/3 astrocytes of strong TG mice by AAV9-hGFAP-RCaMP1.07 (Ohkura et al., 2012). RCaMP in the same astrocytes was repeatedly imaged with varying optical stimulation length (1 mW. 1 s, 3 s or 3 min) before and after systemic retinal injection (Supplementary Figure 2A). We confirmed that the retinal addition is indispensable for Optoα1AR activation even with 3-min illumination (Supplementary Figures 2B–D). With retinal, 1-s illumination was sufficient to elevate the Ca^2+^ levels to saturation, since 3-s illumination only marginally increased the responsive cell number with similar peak amplitudes (Supplementary Figure 2E). Further, 3-min illumination resulted in a single transient Ca^2+^ elevation with a rather smaller peak amplitude (Supplementary Figures 2C,E). In contrast, shorter light activation (1 s or 3 s) was repeatable with an interval of 1 min, although the response magnitude was diminished by about three folds from the second stimulus onwards (Supplementary Figure 2F). Inter-stimulus intervals of 3 and 9 min restored the original response magnitude by 83.4 and 116.0%, respectively (Supplementary Figure 2G).

Thus, we demonstrate that a brief illumination reliably triggers cytosolic Ca^2+^ elevations in Optoα1AR-expressing astrocytes in the presence of cis-retinal and the following experiments were performed with retinal pretreatment by i.p. injection (see methods) unless otherwise noted.

### Astrocytic Gq Signaling Inhibits Neuronal Activity

To investigate the effect of astrocytic Gq activation on neuronal activity, we monitored neuronal Ca^2+^ activities in layer 2/3 of the somatosensory cortex using AAV1.Syn.NES-jRGECO1a.WPRE.SV40 which allows selective expression of the Ca^2+^ probe in neurons (Dana et al., 2016). Brief LED illumination (1 s, 1 mW) in awake TG mice significantly reduced neuronal Ca^2+^ activity quantified as the standard deviation (std) of somatic F/F_0_ jRGECO1a (Figure 2A; Supplementary Video 3). This somatic activity reduction was detectable during the first 1 min (Figure 2C; jRGECO1a F/F_0_ std relative to pre-LED 1 min period: 83.0 ± 4.3%; *p* < 0.008) and continued for an additional minute (Figure 2C; 88.2 ± 3.3%; *p* < 0.02). Similar Ca^2+^ activity reduction was also detected in the neuropil (Figures 2A,C; post-LED 1 min period: 84.1 ± 5.1%; *p* < 0.03; post-LED 1-2 min period: 90.9 ± 3.2%; *p* < 0.03). These suppressive effects could not be attributed to possible photodamage by the two-photon laser or a sensory processing of the LED light, as WT mice did not display such a reduction (Figure 2B; soma, post-LED 1 min period: 98.0 ± 5.0%; *p* > 0.70), which was significantly different from TG (*p* < 0.05, unpaired *t*-test).

Among the molecules that are elevated in the extracellular space after astrocytic activation, adenosine exerts inhibitory effects through the adenosine A1 receptor (A1R) (Pascual et al., 2005; Martin-Fernandez et al., 2017; Tan et al., 2017). When the A1R antagonist, CPT, was applied, this astrocytic Optoα1AR-induced neuronal suppression disappeared and a trend for neuronal activation was observed (Figure 2D; soma post-LED 1 min period: 107.9 ± 5.1%; *p* > 0.18), which was significantly different from that of TG mice without CPT (*p* < 0.004, unpaired *t*-test). These results suggest that transient astrocytic Gq activation rapidly suppresses spontaneous neuronal activity in awake condition *via* A1R. Of note, similar experiments in urethane-anesthetized strong TG mice resulted in a milder decrease of neuronal activity (Supplementary Figure 3A) presumably due to lower basal Ca^2+^ activity (Supplementary Figure 3B).

To examine the effect of astrocytic Gq activation on sensory evoked neuronal activity, we performed *in vivo* field potential (FP) recording from somatosensory cortex layer 2/3 under shallow isoflurane anesthesia (~0.8%) (Figure 2E). After stable FP response for sensory stimulation on the trunk was obtained, brief LED illuminations were delivered from the pial surface above the recording site through optical fiber (φ = 0.2 mm, 1 mW, duration 1 s, interval 5 min, 6 times). As a result, evoked FP slope was decreased after the LED illumination in TG mice, while it was unchanged in WT mice (Figure 2F). This reduction was rapidly expressed and lasting (Figure 2F; 86.7 ± 3.1%, 93.9 ± 3.0%, 84.0 ± 3.6%, and 90.0 ± 3.9% during the first, second, third and fourth 1 min after LED; *p* < 0.008, >0.09, <0.008, and >0.05, paired *t*-test). Furthermore, in the course of a 30-min recording, the reduction of evoked FP slope in TG gradually built up (Figure 2G; 89.4 ± 2.0% and 82.3 ± 6.5% during 20–25 min and 25–30 min after the first LED; *p* < 0.004 and <0.05, paired *t*-test). These results suggest that transient astrocytic Gq activation inhibits evoked synaptic response and repeated astrocytic Gq activations lead to synaptic depression.

### Behavioral Impact of Astrocytic Gq Signaling

Although multiple studies show that noradrenergic Gq signal simultaneously activates astrocytic Ca^2+^ elevation in wide cortical regions (Bekar et al., 2008; Ding et al., 2013; Paukert et al., 2014), its *in vivo* roles remain unknown. To understand how global astrocytic Gq signaling affects mouse behavior, we performed a set of behavioral experiments with Optoα1AR TG mice whereby photostimulation was made using a head-mounted wireless LED device (Iwai et al., 2011; Hashimoto et al., 2014). A blue LED (φ = 3.1 mm) was placed on each side of the thinned skull above the anterior cortex, where dense noradrenergic inputs are projected in the upper layers (Agster et al., 2013).

A single exposure of blue light for 3 s to wide anterior cortical areas (AP ~1.5 mm, ML ~1.5 mm) did not result in obvious immediate behavioral changes in strong TG mice. For instance, there was no sign of arousal from sleep or falling to sleep by the optical stimulation. We next investigated the exploratory behavior of mice in a novel open field for 45 min while intermittently illuminating the anterior cortical areas (duration 3 s, interval 3 min, 15 times, for both WT and TG; Figure 3A). As demonstrated in a single-animal example in Figure 3A, the TG mouse had a sign of lower exploratory behavior than the WT mouse. To check the level of anxiety, time in center domain was quantified, and there was not a significant difference between WT and TG mice in any of the trichotomized time intervals (Figure 3B). Locomotion was consistently lower in optically stimulated TG mice throughout the course of the open field test and the difference from WT mice became more significant in the middle and final 15 min periods (Figures 3C,D). Analysis of immobile time shows that TG mice gradually develop immobility and the difference from WT mice becomes distinct in the last 20 min period (Figure 3E).

Time-averaged analysis of locomotion with respect to LED illumination indicates that the transient astrocytic Gq activation reduced the locomotion activity rapidly. This reduced activity continued until the next astrocytic Gq activation (Figure 3F). Therefore, the astrocytic Gq signal-triggered reduction of locomotion accumulated at every LED illumination, resulting in larger locomotion differences detected in later periods (Figures 3C,D). Notably, when the A1R antagonist DPCPX was applied at the dosage that does not affect open field locomotion in WT mice (Griebel et al., 1991; Sun et al., 2016) (1 mg/kg), the reduced locomotion in the strong TG mice by LED illumination was reverted (Figures 3D,F). Patchy TG mice showed a similar trend for reduced locomotion activity, although the degree of reduction was smaller (Supplementary Figure 4), suggesting that thorough activation of astrocytic Gq signaling is required for the decreased locomotion. Consistently, insufficient Gq activation without retinal supply could not induce the locomotion decrease (Supplementary Figure 5).

Next, we examined the influence of astrocytic Gq signaling on memory. Anterior cortical areas including the prefrontal cortex have been shown to regulate memory acquisition and maintenance (Simons and Spiers, 2003; Frankland and Bontempi, 2005; Euston et al., 2012), which could be mediated by noradrenergic input (Arnsten et al., 2012). To evaluate the involvement of astrocytic Gq activation in working memory, we performed the Y-maze test. Both strong TG and WT mice freely explored in the Y-maze for 15 min while receiving blue LED stimulation (duration 3 s, interval 3 min, 5 times; Figure 4A). The probability of the correct arm entry (i.e., the entry to the arm different from the current and immediate prior ones) did not change significantly between TG and WT mice, although its variability was higher in TG mice (Figure 4B). The number of arm entries, total distance traveled and immobile time were also similar between TG and WT mice (Figures 4C–E). These results indicated that the transient astrocytic Gq signal activation in anterior cortical areas does not affect working memory or alter the short-term exploratory behavior in the Y-maze.

We then tested long-term memory by performing the novel object recognition test. On day 1, anterior cortical areas were illuminated with LED (duration 3 s, interval 3 min, four times) during the object familiarization period (training period), whereby two identical objects located apart were exposed for 10 min in a behavior chamber. When one of the pre-exposed objects was replaced with a novel object 1 day later (Figure 5A, left), WT and strong TG mice similarly spent a longer time contacting the novel object relative to the familiar object (Figure 5A, middle). Notably, when object replacement was done 14 days later, TG mice still retained the novel object preference, whereas WT mice lost the preference (Figure 5A, right). Even if the duration of each LED illumination was increased from 3 s to 30 s (Figure 5B), TG mice showed a similar novel object preference 14 days later, suggesting that 3 s activation is sufficient to achieve the plateaued enhancement in object memory retention. Of note, LED illumination of TG mice without retinal supply could not significantly induce this 14-day memory retention (Figure 5C), consistent with insufficient Gq activation. Moreover, LED illumination of TG mice with DPCPX treatment did not result in 14-day memory retention, although the novel object recognition after 1 day was normal (Figure 5D). These experiments suggest that transient activation of astrocytic Gq signal does not affect memory acquisition but enhances memory that lasts for more than two weeks through A1R activation.

Previous studies showed that systemic injection of noradrenaline (NA) immediately after training enhances long-term object recognition memory (Dornelles et al., 2007; Jurado-Berbel et al., 2010). To test if astrocytic Gq activation induces similar effects, we photo-stimulated TG mice immediately after training (duration 3 s, interval 3 min, four times; Supplementary Figure 6A) and found that this post activation was also effective for memory enhancement 14 days later (Supplementary Figure 6A, right). Unlike prolonged exposure to an open environment (Figure 3), neither locomotion nor object contact time differed significantly during the 10 min training period across genotypes, illumination durations or drug applications (Supplementary Figures 6B,C). Furthermore, the memory-enhancing effect is not due to an avoidance behavior from familiarized objects which were associated with astrocytic Gq activation, as the conditioned place preference test suggests that astrocytic Gq activation did not change environmental preference to activated side or non-activated side (Supplementary Figure 7).

## Discussion

The identification of the optogenetic stimulation conditions for the Optoα1AR TG mice allowed causal assessment of physiological Gq signaling in astrocytes for the first time. Optogenetic activation of cortical astrocytic Gq signaling transiently inhibited local neuronal activity *via* the adenosine A1 receptor, induced depression of evoked response when paired with sensory stimuli, and reduced locomotor activity. Furthermore, transient astrocytic Gq signaling resulted in the enhancement long-term remote memory (novel object recognition test), while short-term memory (STM, Y-maze test) was not affected.

### Astrocytic Gq Signaling-Induced Neuronal Inhibition

Effects of astrocytic Gq activation on neuronal activity remains controversial. For instance, in a recent pharmacogenetic study (Adamsky et al., 2018) and an optogenetic study (Mederos et al., 2019), astrocytic Gq activation induced long-term potentiation (LTP) in hippocampal CA1 slices, but an earlier pharmacogenetic study reported no effects (Agulhon et al., 2010). Whereas these studies did not report *in vivo* effects on astrocytic and neuronal activity, we showed rapid reduction of neuronal activity and depression of evoked response after astrocytic Gq activation (Figure 2), in line with the recent monkey prefrontal cortex study with α1AR agonists (Datta et al., 2019). Aside from the preparation differences, one important aspect of the current study is the use of TG mice that minimizes concerns on tissue inflammation caused by virus-injection and slice preparation. Indeed, acute astrogliosis alters multiple plasticity-related molecules (Takano et al., 2014), which potentially impact neuronal activity through reactive astrocytes (Agulhon et al., 2012; Liddelow et al., 2017). Our immunohistochemical examination by GFAP and Iba1 shows that astrocytes and microglia appear healthy with little sign of inflammation: overall, GFAP immunoreactivity is low in the cortex, and even when GFAP immunoreactivity is apparent, the intracellular appearance is modest. Likewise, Iba1 pattern shows a fine ramified morphology of non-inflammatory microglia (Supplementary Figures 1, 8).

Cholinergic activation elevates astrocytic Ca^2+^ and promotes cortical and hippocampal synaptic potentiation when combined with sensory stimulation in anesthetized rodents (Takata et al., 2011; Chen et al., 2012; Navarrete et al., 2012). Moreover, noradrenergic activation *via* transcranial direct current stimulation induces synaptic potentiation in the cortex (Monai et al., 2016). The apparent difference of plasticity expression may be explained by the manner whereby GPCR is activated. Volume-transmitted neuromodulators act on neuronal and astrocytic GPCRs, both of which may be required for the synaptic potentiation. Likewise, these neuromodulators also activate Gs and/or Gi/o signaling, that may also play an essential role (Hirase et al., 2014). Importantly, the current study selectively stimulates astrocytic Gq signaling in a temporally defined manner, thereby dissecting specific roles of the signaling in the neural circuit.

Our results indicate that the astrocytic Gq-activated neuronal inhibition involves adenosine signaling. Previous work has documented astrocyte-derived synaptic activity of the adenosine A1 receptor *in vitro* (Zhang et al., 2003; Pascual et al., 2005; Panatier et al., 2011) and such signaling is suggested to take place in *in vivo* under certain conditions (Florian et al., 2011; Li et al., 2020). Astrocytic release of ATP, by exocytosis or through channels, has been suggested to be a source of adenosine (Butt, 2011, for a review). At a revision of this manuscript (Iwai et al., 2019), we investigated the ATP-mediated responses of Optoα1AR-negative astrocytes with the broadband P2 receptor blocker PPADS. We indeed observed that the activation of Optoα1AR-negative astrocytes is largely suppressed (Supplementary Figure 9A). Moreover, the onset of Ca^2+^ elevation in activated Optoα1AR-negative astrocytes are longer (Supplementary Figure 9B). These results suggest a role of purinergic signaling in the delayed activation of Optoα1AR-negative astrocytes. However, we also saw weak YFP signals in some Optoα1AR-negative astrocytes after immunohistochemical enhancement by a polyclonal GFP antibody (Supplementary Figure 8D), indicating a possibility that the delayed activation of Optoα1AR-“negative” astrocytes could be due to activation of the faintly expressed Optoα1ARs. A further study is needed to address this issue.

### Astrocytic Gq Signaling-Induced Behavioral Changes

We found that transient astrocytic Gq activation in wide anterior cortical areas gradually attenuated locomotion in a novel open field (Figure 3), which is consistent with its neuronal suppressive effects (Figure 2). While noradrenergic activation is generally thought to promote arousal, a study showed that LC noradrenergic neuronal discharge pattern modulates locomotor activity: Phasic high-frequency bursts decrease locomotor activity, and tonic low-frequency discharges increase locomotion (Carter et al., 2010). These modes of LC discharge may activate distinct noradrenergic receptors: tonic discharge might preferentially activate the high-affinity α2AR, whereas phasic discharge may additionally activate the low-affinity α1AR and βARs. A previous pharmacogenetic study of systemic astrocytic Gq activation reported reduced locomotor activity and proposed that sustained Gq signaling in the autonomic nervous system might underlie this effect (Agulhon et al., 2013). Our study indicates that transient astrocytic Gq activations in the anterior cortical area, which includes motor areas, are sufficient to reduce locomotion presumably by inhibition of the neuronal activity.

### Astrocytic Gq Signaling-Induced Long-Term Memory Enhancement

The absence of effects on STM by the astrocytic Gq signal activation in our study (Figure 4) contrasts with earlier studies that showed an impairment of spatial STM by infusion of α1AR agonists in the prefrontal cortex (Arnsten et al., 1999; Birnbaum et al., 2004) or an enhancement of STM in T-maze by pharmacogenetic activation of hippocampal CA1 (Adamsky et al., 2018). This inconsistency could be due to a combination of multiple factors including activation area (global anterior cortex vs. local hippocampal CA1), activation mode (transient vs. sustained), and astrocytic Ca^2+^ response magnitude. For the last point, our optogenetic activation results in Ca^2+^ elevations similar to natural astrocytic responses (Figure 1), whereas other studies did not address this issue.

We showed that the astrocytic Gq signal activation did not affect object recognition memory retrieval 1 day after learning, whereas it enhanced memory 2 weeks after (Figure 5). In contrast, the pharmacogenetic astrocytic Gq signal activation has been reported to enhance 1-day memory in a contextual fear conditioning task (Adamsky et al., 2018). Adamsky and colleagues also showed that continuous 5-min illumination of astrocytic Optoα1AR (90% duty cycle) similarly induced a 1-day memory enhancement; however, comparison with our result is difficult due to the lack of description for *in vivo* astrocytic Ca^2+^ elevation and cis-retinal supplement, aside from the learning-paradigm difference. Another recent study showed that 3-min illumination of melanopsin expressed in CA1 astrocytes induced a memory enhancement in an object place test probed 30-min after association (Mederos et al., 2019). However, direct comparison to our result is difficult due to the lack of *in vivo* activation data as well as the concern that the melanopsin (Opn4-human) used in the study activates the Gi/o pathway in addition to the Gq pathway (Bailes and Lucas, 2013). Our study highlights that transient astrocytic Gq activation promotes remote memory that lasts over weeks and is in line with Lee et al. (2014) that reported impairment of 2-week object recognition memory in mice deficient of astrocytic vesicular release. However, the origin of extracellular adenosine remains controversial. While previous studies have described vesicular release of adenosine by astrocytes (Pascual et al., 2005; Schmitt et al., 2012), other studies have demonstrated neuronal release of adenosine (Lovatt et al., 2012; Wu et al., 2020). The exact mechanism that links to astrocytic Gq signaling to extracellular adenosine increase should be addressed in future studies.

### Astrocytic Gq Signaling-Mediated Novelty Detection

Long-term memory (LTM) enhancement by brief astrocytic activation during or shortly after object familiarization (Figure 5; Supplementary Figure 6) is reminiscent of previous studies whereby NA was systemically administered immediately after object familiarization (Dornelles et al., 2007; Jurado-Berbel et al., 2010). These results are in line with the “behavioral tagging” hypothesis (Moncada and Viola, 2007; Ballarini et al., 2009), which is a behavioral analog of the synaptic tagging and capture theory (Frey and Morris, 1997; Redondo and Morris, 2011): ordinary training sets tags on relevant synapses, and a novel experience induces the synthesis of plasticity-related proteins (PRPs) that are captured at the tagged synapses for memory consolidation (Supplementary Figure 10). Previous studies have suggested that dopaminergic and noradrenergic inputs are required for hippocampus-dependent LTM (Wang et al., 2010; Moncada et al., 2011), which are likely activated by co-release of the two neuromodulators from LC axons (Kempadoo et al., 2016; Takeuchi et al., 2016).

We propose that astrocytic Gq signaling mediates the effect of novel experience to promote the persistence of long-term memory (Supplementary Figure 10). For instance, LC noradrenergic neurons fire phasically in response to novel and salient events (Aston-Jones and Bloom, 1981; Vankov et al., 1995) and high amounts of released NA in turn trigger astrocytic Gq signaling *via* α1ARs (Ding et al., 2013; Paukert et al., 2014). Other subcortical neuromodulators such as acetylcholine and dopamine as well as activation of Gi/o-coupled GPCR, which also induce IP_3_-dependent Ca^2+^ signaling (Kofuji and Araque, 2020), may also be involved. Among possible PRPs for LTM, including Homer1a, Arc, BDNF and PKMζ (protein kinase Mζ), a recent study indicated that PKMζ in prefrontal cortex serves as a PRP for object recognition memory (Naseem et al., 2019). Although astrocytic Gq signaling has yet been shown to induce PRPs in neurons, elevated adenosine A1R signaling in neurons is known to increase ERK-phosphorylation and Hormer1a expression *in vivo* (Serchov et al., 2015). ATP release from astrocytes and the subsequent conversion to adenosine conceivably induce PRPs *via* adenosine A1R (① in Supplementary Figure 10).

Synaptic depression has been suggested as a key mechanism for novelty detection and memory (Manahan-Vaughan and Braunewell, 1999; Griffiths et al., 2008; Dong et al., 2012). NA application to acute brain slices induced long-term depression (LTD) in an α1AR-dependent manner (Kirkwood et al., 1999; Scheiderer et al., 2004), which is presumably mediated by astrocytic ATP release (Pougnet et al., 2014). Moreover, phasic stimulations of LC *in vivo* induced βAR-dependent LTD in the hippocampus (Lemon et al., 2009). Notably, serum response factor (SRF)-deficient mice are impaired of hippocampal LTD and do not habituate to novel environments (Etkin et al., 2006). Our results of synaptic depression and reduced locomotion by astrocytic Gq activation (Figures 2, 3) complement these phenotypes and further postulate that astrocytic Gq activation may be involved in SRF signaling. Indeed, the α1AR has been shown to activate SRF in non-neuronal cells (Hennenberg et al., 2012). Of particular note, a recent study reported LTD deficiency in IP_3_R2-KO mice (Pinto-Duarte et al., 2019), which are deficient of large cytosolic Ca^2+^ surges in astrocytes. Together, these findings lend support to the notion that novelty triggers astrocytic Gq signal and induces synaptic depression. Such a mechanism may underlie novelty-acquisition behaviors such as habituation to a novel environment. How LTD facilitates STM-to-LTM conversion remains unknown. It is tempting to speculate that LTP of relevant synapses for memory formation carries relatively high information under the LTD-favored condition, due to an improved signal-to-noise ratio.

In the current work, we activated Gq signaling in a large population of astrocytes to mimic astrocytic activation by volume-transmitted neuromodulators, which induces large IP_3_-dependent Ca^2+^ elevations from the endoplasmic reticulum. It is noted that astrocytes can also increase intracellular Ca^2+^ levels *via* other mechanisms, for example, through reversed sodium-calcium exchangers in fine (peri-synaptic) processes (Rose et al., 2020). It is conceivable that Ca^2+^ elevations by extracellular Ca^2+^ influx and by internal release from subcellular organelles serve distinct and/or synergistic functions (Semyanov, 2019).

On a final note, a very recent study has reported that astrocytic Gi-GPCR signaling by Gi-DREADD enhances remote fear memory (Kol et al., 2020). Since astrocytic Gi-GPCRs (e.g., GABA-B or CB1 receptors) also give rise to IP3-mediated Ca^2+^ elevation, astrocytic Ca^2+^ signaling is conceivably involved in this process. Our study demonstrated that transient Ca^2+^ signaling as seen during fear conditioning (Oe et al., 2020), is sufficient for the enhancement of remote memory.

## Data Availability Statement

The raw data supporting the conclusions of this article will be made available by the authors, without undue reservation.

## Ethics Statement

The animal study was reviewed and approved by the Institutional Animal Care and Use Committee of RIKEN and the Danish Animal Experiments Inspectorate.

## Author Contributions

YI, KO, HH, and SI: study conception and design. YI, KO, KY, TM, SA, CV, AL, and MT: data collection. YI, KO, TM, SA, CV, AL, MT, and HH: analysis and interpretation. YI, KO, and HH: draft manuscript preparation. All authors reviewed the results and approved the final version of the manuscript.

## Conflict of Interest

The authors declare that the research was conducted in the absence of any commercial or financial relationships that could be construed as a potential conflict of interest.
