# Supplementary material for: Transient Astrocytic Gq Signaling Underlies Remote Memory Enhancement
Source: Front Neural Circuits. 2021 Mar 22;15:658343. doi: 10.3389/fncir.2021.658343 (PMC8019746; doi:10.3389/fncir.2021.658343)
Supplement: Supplementary file 1 [file Data_Sheet_1.PDF]

# Transient astrocytic Gq signaling underlies remote memory enhancement

## *Supplementary materials*

Youichi Iwai[1+], Katsuya Ozawa[1+], Kazuko Yahagi[1+], Tsuneko Mishima[2+], Sonam Akther[2], Camilla Trang Vo[2], Ashley Bomin Lee[2], Mika Tanaka[1,3], Shigeyoshi Itohara[3], Hajime Hirase[1, 2*]

1. Laboratory for Neuron-Glia Circuitry
RIKEN Center for Brain Science
Wako, Saitama, Japan

2. Center for Translational Neuromedicine
Faculty of Medical and Health Sciences,
University of Copenhagen
Copenhagen, Denmark

3. Laboratory for Behavioral Genetics
RIKEN Center for Brain Science
Wako, Saitama, Japan

## List of supplementary materials

**Supplementary Figure S1**: “Strong” and “Patchy” TG mouse lines have astrocyte-selective Optoα1AR-EYFP (OptoA1AR-EYFP) expression without inflammation.

**Supplementary Figure S2**: Brief illumination of Optoα1AR fully and repeatably activates astrocytic $Ca^{2+}$ elevation only after retinal addition.

**Supplementary Figure S3**: Brief astrocytic Gq activation under urethane-anesthesia did not significantly suppress spontaneous neuronal activity.

**Supplementary Figure S4**: Patchy TG mice show a trend of decreased open-field activity during LED activations.

**Supplementary Figure S5**: Decreased open-field locomotion activity in strong TG mice by LED activations depends on retinal addition.

**Supplementary Figure S6**: Astrocytic Gq activation immediately after training enhances long-term memory, and exploratory behaviors during training is not related to memory effects.

**Supplementary Figure S7**: Anterior cortical astrocytic Gq activation does not affect conditioned place preference.

**Supplementary Figure S8**: Confocal images of the “Patchy” TG line with cell-type specific markers in the cerebral cortex.

**Supplementary Figure S9**: Purinergic receptor blockade results in diminishment of delayed activation in Optoα1AR-negative astrocytes in the “Patchy” TG line.

**Supplementary Figure S10**: Proposed model for astrocytic Gq signaling-mediated enhancement of long-term memory in novelty detection.

**Supplemental Video 1**: Astrocytic $Ca^{2+}$ imaging upon brief Optoα1AR activation by weak LED illumination, corresponding to the Figure 1F.

**Supplemental Video 2**: Astrocytic $Ca^{2+}$ imaging upon brief Optoα1AR activation by strong LED illumination, corresponding to the Figure 1G.

**Supplemental Video 3**: Neuronal $Ca^{2+}$ imaging upon brief Optoα1AR activation, corresponding to Figure 3A.

## Supplementary Figure S1

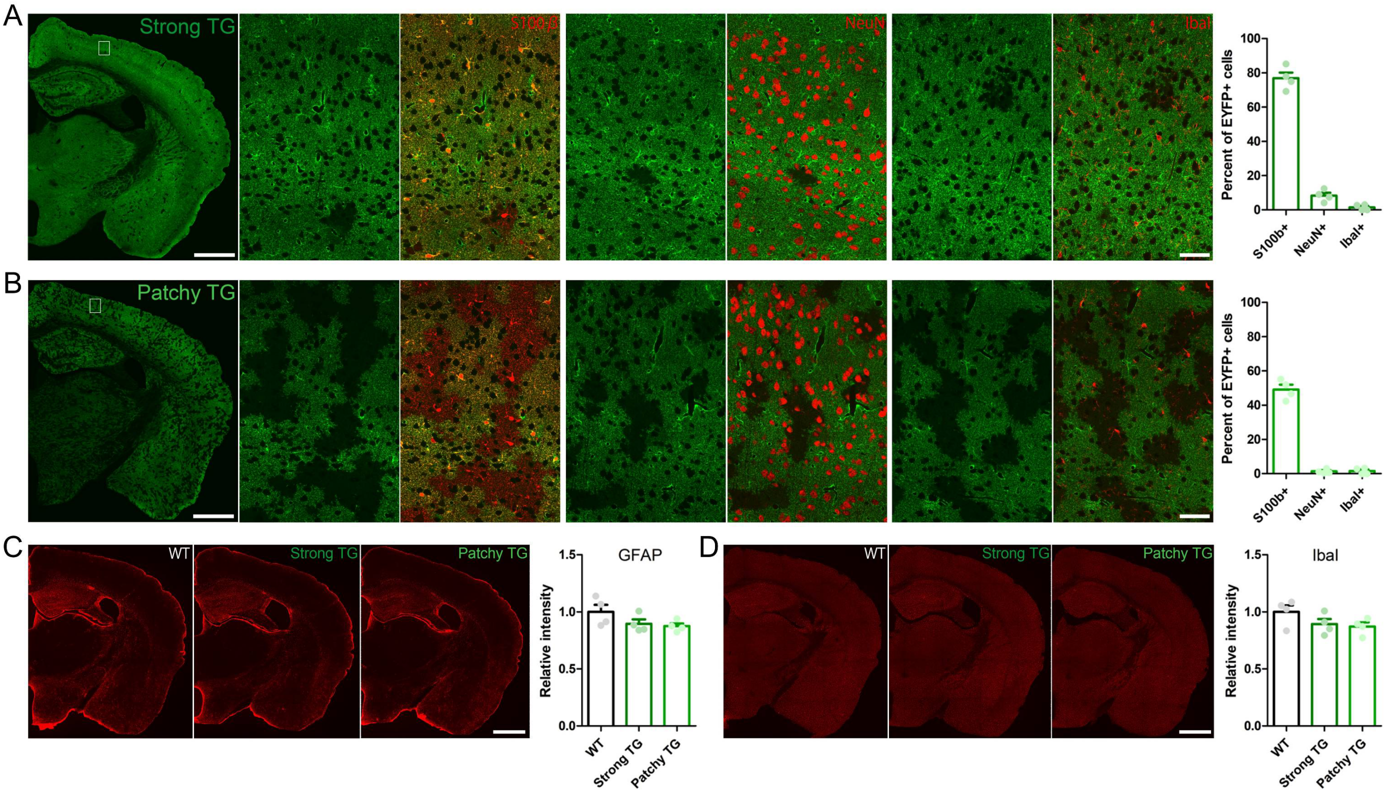


**Supplementary Figure S1**: "Strong" and "Patchy" TG mouse lines have astrocyte-selective Optoα1AR-EYFP expression without inflammation.

(A and B) EYFP fluorescence images corresponding to the S100β and NeuN images in **Figures 1B,C** are shown. Adjacent sections are also immunolabeled by microglial specific marker, Iba1. Among the cellular marker-positive cells, percent of EYFP-positive cells were quantified. Scale bar: 1 mm (left), 50 μm (the rest).

(C) GFAP immunoreactivity of WT, Strong TG, and Patchy TG mice. Cortical GFAP signals are weak and similar across the genotypes (somatosensory cortex: $p>0.15$, one-way ANOVA, 4 WT, 4 strong TG vs 4 patchy TG mice). Scale-bar: 1 mm.

(D) Iba1 immunoreactivity of WT, Strong TG, and Patchy TG mice. Iba1 signals are not increased in either of TG mouse lines (somatosensory cortex: $p>0.16$, one-way ANOVA, 4 WT, 4 strong TG vs 4 patchy TG mice). Scale-bar: 1 mm.

Micrographs in C and D are further magnified in the following page.

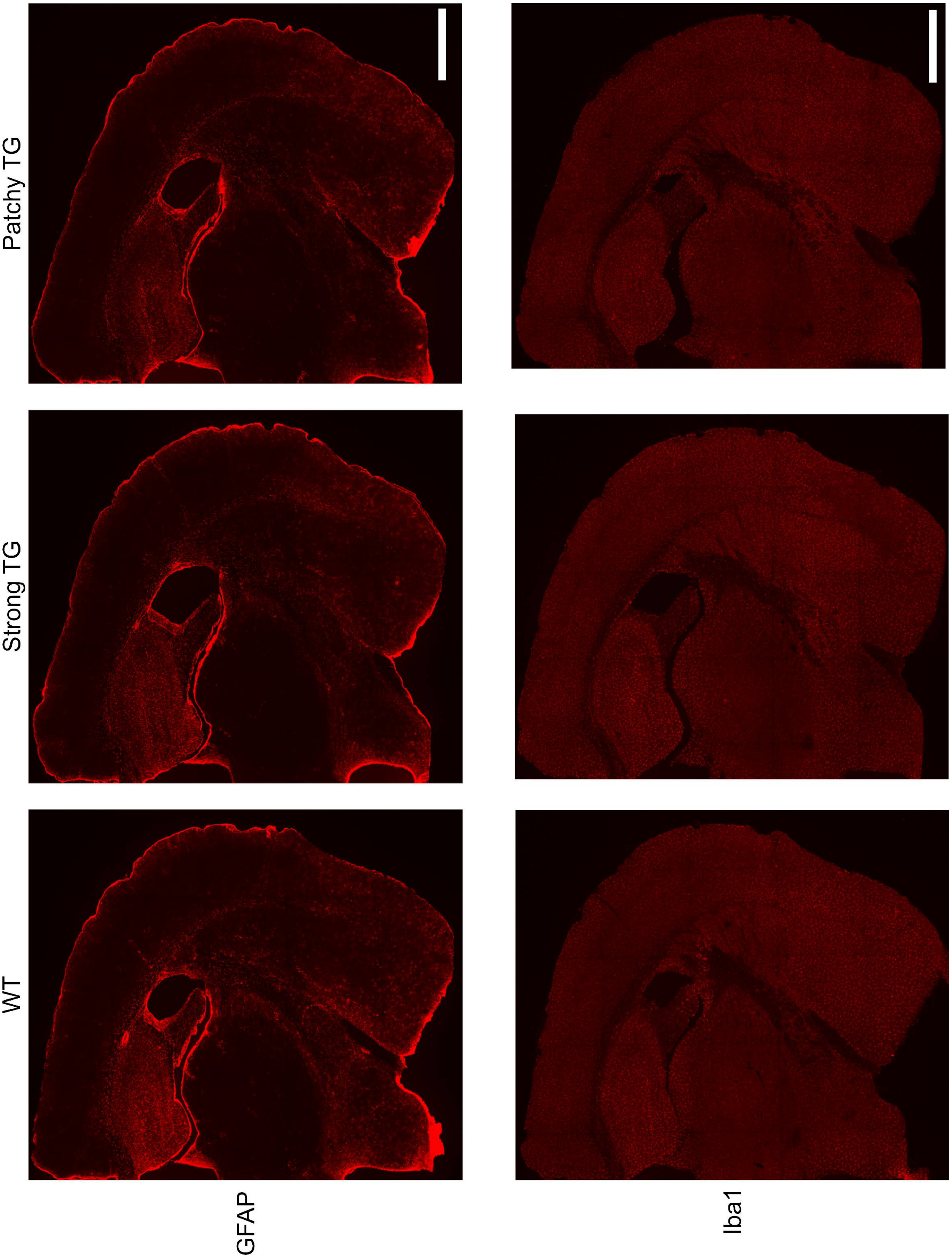
Patchy TG
Strong TG
WT
GFAP
Iba1

## Supplementary Figure S2

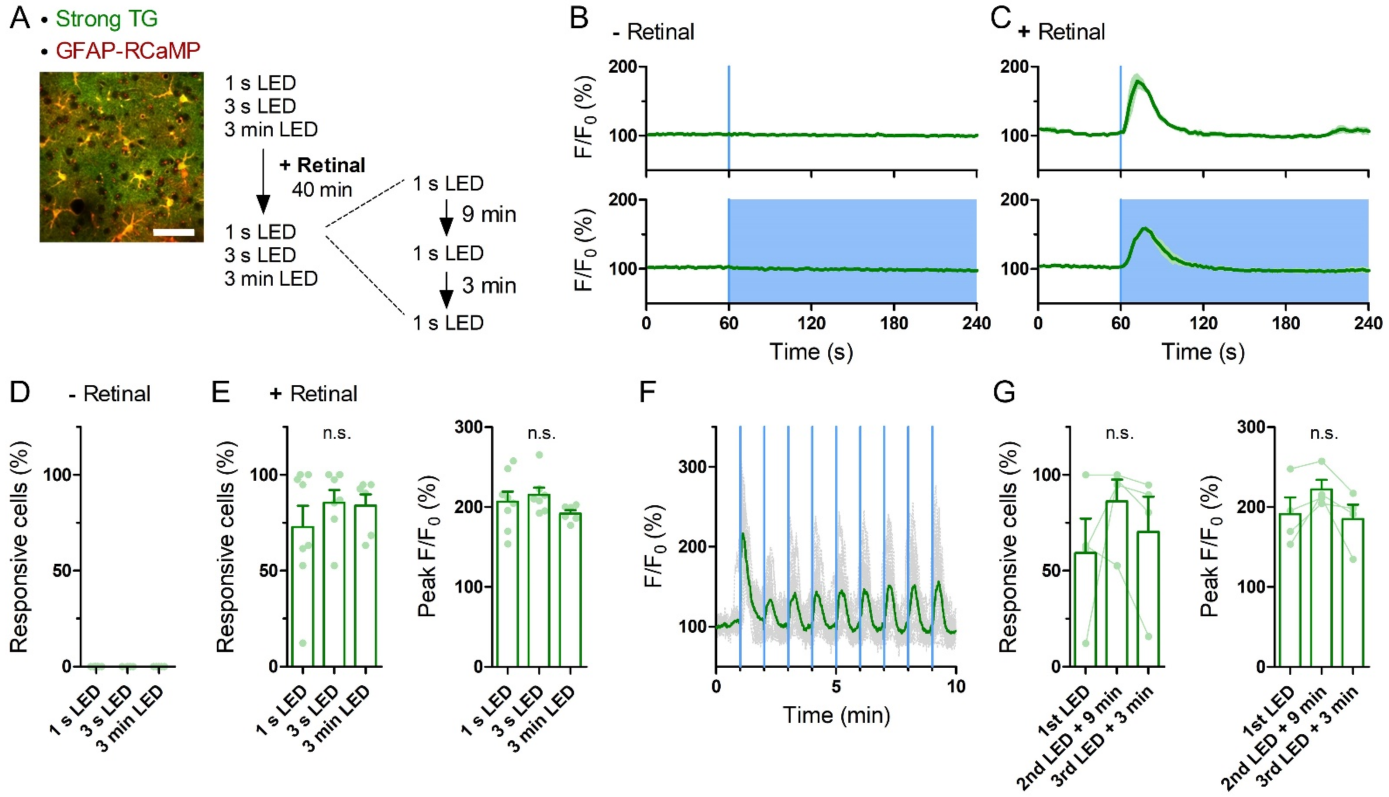


**Supplementary Figure S2**: Brief illumination of Optoα1AR fully and repeatably activates astrocytic $Ca^{2+}$ elevation only after retinal addition.

(A) Representative two-photon image of somatosensory cortex layer 2/3 of a urethane-anesthetized strong TG mouse with AAV-induced RCaMP expression in astrocytes (left). RCaMP signal was imaged with LED illuminations (1 mW) of varying durations (1 s, 3 s or 3 min) before and after retinal addition (i.p.). Scale bar: 50 µm.

(B and C) $Ca^{2+}$ response of Optoα1AR-positive astrocytes upon 1-s or 3-min illumination (top and bottom, respectively), before and after retinal injection. 1 s or 3 min illumination failed to activate $Ca^{2+}$ signaling before retinal injection. After retinal injection, 1-s illumination induced a transient $Ca^{2+}$ elevation. The peak amplitude of $Ca^{2+}$ elevation was similar for 3-min illumination, suggesting that the brief illumination induces the saturated amplitude of $Ca^{2+}$ elevation. Small SEM (light-green) indicates a reproducible response.

(D) Before retinal injection, $Ca^{2+}$ response was not detected in astrocytes upon 1 s, 3 s or 3 min illumination. Each symbol represents an individual imaging session (N=7, 4 and 5 sessions from 3 strong TG mice for 1 s, 3 s and 3 min illuminations, respectively).

(E) After retinal injection, responsive cells and peak $F/F_0$ were observed upon illumination. These parameters were similar among varying illumination durations ($p>0.51$ and $p> 0.14$, one-way ANOVA and Kruskal-Wallis one-way ANOVA). Each symbol represents an individual imaging session (N=8, 7 and 6 from 3 strong TG mice for 1 s, 3 s and 3 min illuminations, respectively).

(F) Demonstration of repeatability of Optoα1AR stimulation. One-minute interval results in a notable attenuation of $Ca^{2+}$ response. Individual and mean $Ca^{2+}$ responses upon 9 times LED illuminations were indicated by gray and green traces, respectively (26 astrocytes).

(G) After retinal injection, imaging was performed at intervals of 9 min and 3 min as indicated in A. Proportion and response amplitude of responsive cells were similar for both inter-stimulus intervals (9 min: $p>0.29$ and $p>0.07$, paired t-test, 4 pairs; 3 min: $p>0.12$ and $p>0.09$, paired t-test, 4 pairs). As the values induced by the 1st and 2nd illuminations were not significantly different, these were mixed and presented in (E).

## Supplementary Figure S3

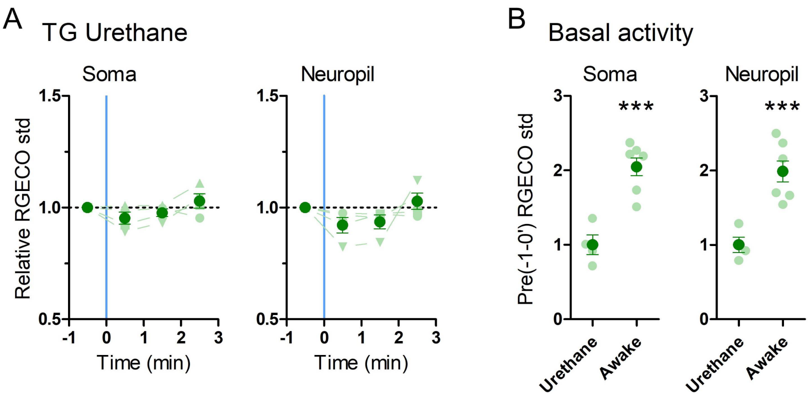


**Supplementary Figure S3**: Brief astrocytic Gq activation under urethane-anesthesia did not significantly suppress spontaneous neuronal activity.

(A) Relative $Ca^{2+}$ activities of neuronal somata and neuropil are calculated as the standard deviation (std) of RGECO $F/F_0$ in urethane-anesthetized strong TG mice. Somata or neuropil $Ca^{2+}$ activities were not affected by LED illumination under urethane-anesthesia (soma post-LED 1-min period: 95.3±2.7%; p>0.17, paired t-test vs pre-LED 1-min period; neuropil post-LED 1-min period: 92.2±3.4%; p>0.10, paired t-test vs pre-LED 1-min period, 4 mice).

(B) Baseline $Ca^{2+}$ activities of neuronal somata and neuropil were visibly lower in the urethane-anesthetized condition than in awake conditions (soma awake pre-LED 1-min period: 204.8±11.9% relative to soma urethane pre-LED 1-min period; p<0.001, unpaired t-test; neuropil awake pre-LED 1-min period: 198.8±13.9% relative to neuropil urethane pre-LED 1-min period; p<0.001, unpaired t-test).

## Supplementary Figure S4

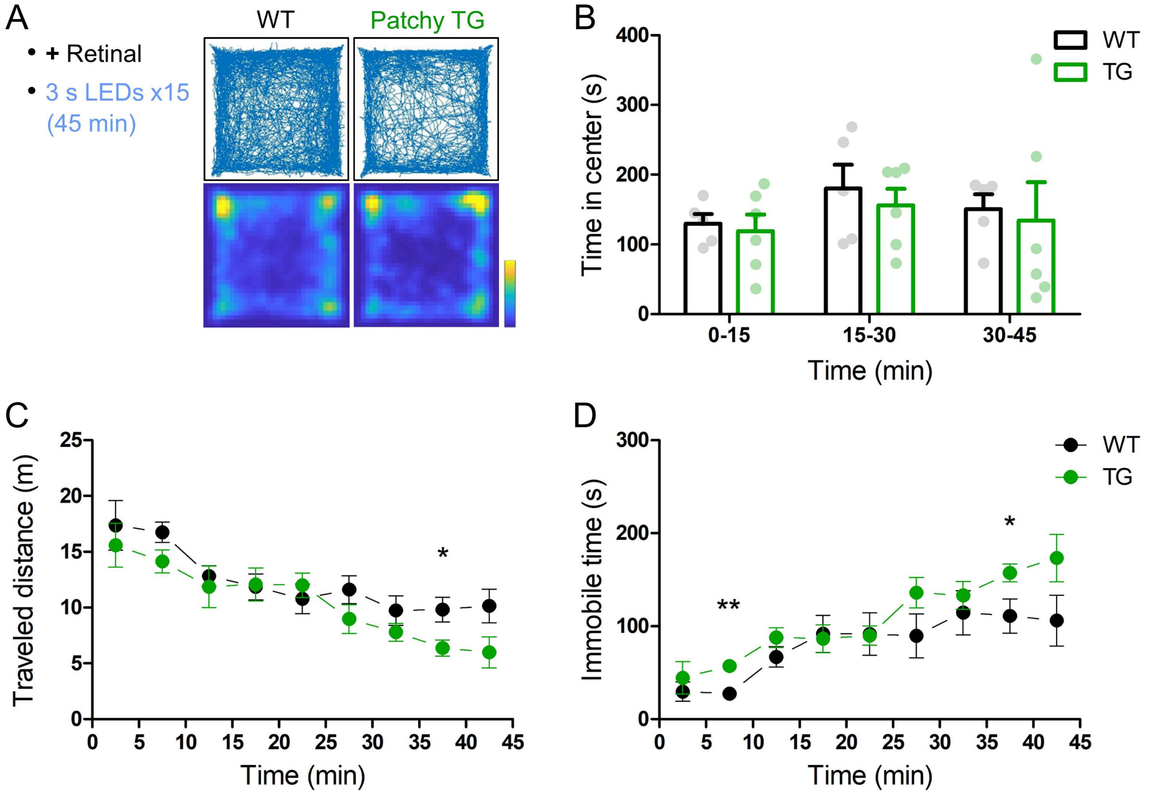


**Supplementary Figure S4**: Patchy TG mice show a trend of decreased open-field activity during LED activations.

(A) Novel open field test of WT and patchy TG mice during transient LED illuminations (duration 3 sec, interval 3 min, 15 times) with retinal pre-treatment. Representative locomotion traces and time maps for 45 min show that the TG mouse traveled shorter distances, while spending a similar length of time in the center zone as the WT mouse. Color bar: 15 s.

(B) Time in center zone was not significantly different between genotypes and between 15 min periods ($p>0.53$ and $p>0.42$, two-way ANOVA, 5 WT mice vs 6 patchy TG mice).

(C) TG mice tended to travel a shorter distance (*$p<0.05$, unpaired t-test, 5 WT mice vs 6 patchy TG mice).

(D) TG mice tended to have increased immobile time (**$p<0.01$, *$p<0.05$, unpaired t-test, 5 WT mice vs 6 patchy TG mice).

## Supplementary Figure S5

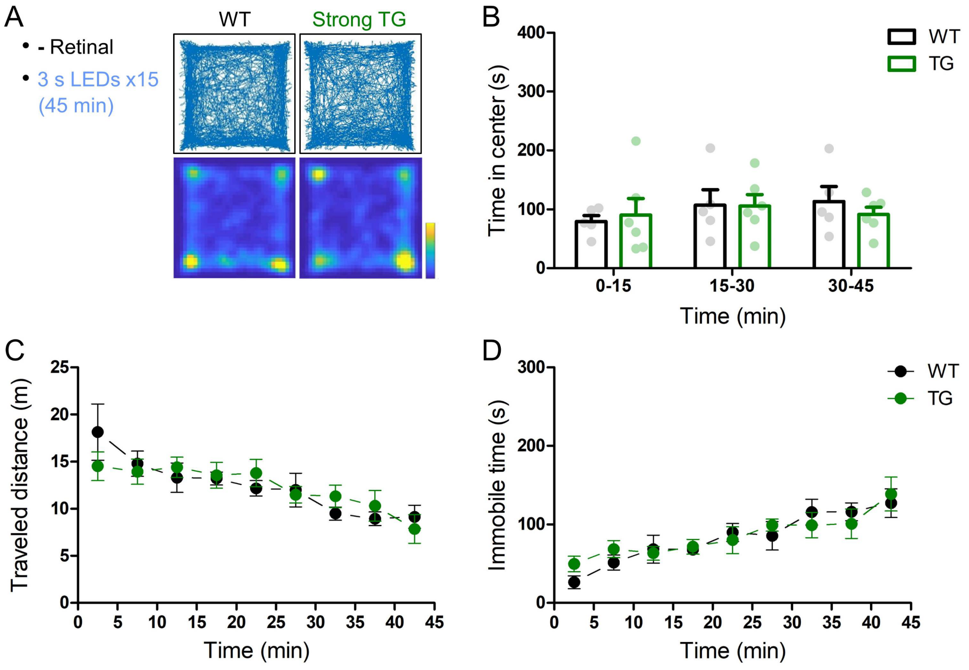


**Supplementary Figure S5**: Decreased open-field locomotion activity in strong TG mice by LED activations depends on retinal addition.

(A) Novel open field test of WT and strong TG mice during transient LED illuminations (duration 3 sec, interval 3 min, 15 times) with pre-treatment of vehicle instead of retinal. Representative locomotion traces and time maps for 45 min show that the TG mouse traveled similar distance and spent similar time in the center zone as the WT mouse. Color bar: 15 s.

(B) Time in center zone was not significantly different between genotypes and between 15 min periods ($p>0.81$ and $p>0.57$, two-way ANOVA, 5 WT mice vs 6 strong TG mice).

(C) TG mice traveled similar distance as WT mice ($p>0.24$, unpaired t-test, 5 WT mice vs 6 strong TG mice).

(D) TG mice had similar immobile time as WT mice ($p>0.11$, unpaired t-test, 5 WT mice vs 6 strong TG mice).

## Supplementary Figure S6

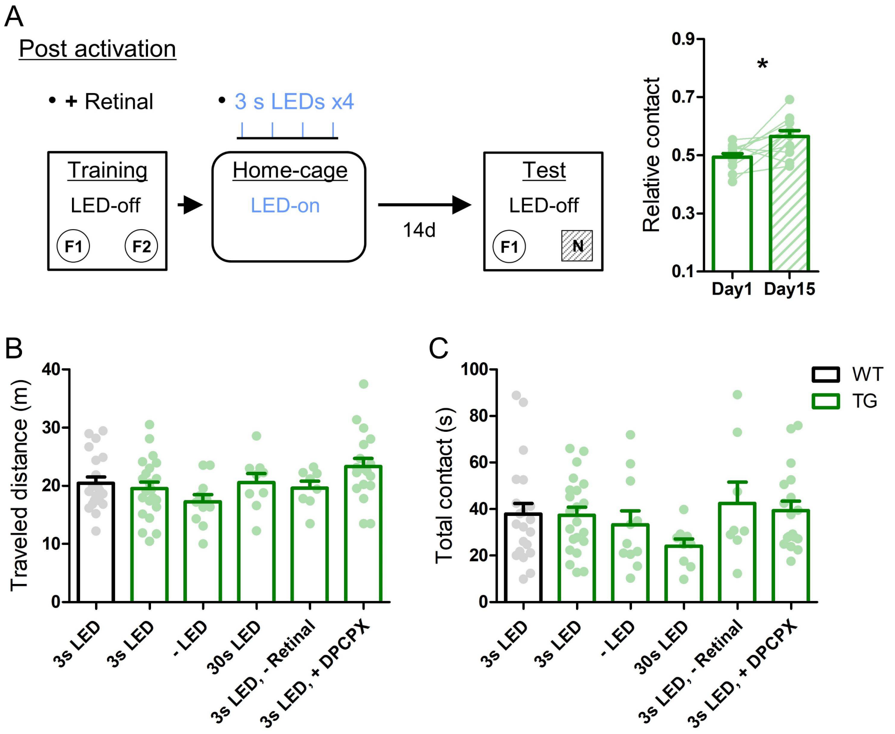


**Supplementary Figure S6**: Astrocytic Gq activation immediately after training enhances long-term memory, and exploratory behaviors during training is not related to memory effects.

(A) Strong TG mice received LED illuminations (duration 3 s, interval 3 min, 4 times) in the home cage immediately after training. This post-activation protocol also induced the novel object preference 14 days after training ($p<0.02$, paired t-test, 11 strong TG mice).

(B) Distance traveled during training was not significantly different between genotypes, between LED durations or between drug treatments ($p>0.54$, unpaired t-test, 21 WT mice with 3 s LED vs 22 TG mice with 3 s LED; $p>0.28$, one-way ANOVA, 22 TG mice with 3 s LED, 11 TG mice without LED vs 9 TG mice with 30 s LED; $p>0.06$, one-way ANOVA, 22 TG mice with 3 s LED, 8 TG mice with 3 s LED and without retinal vs 18 TG mice with 3 s LED and with DPCPX).

(C) Total contact time with both objects during training was not significantly different between genotypes, between LED durations or between drug treatments ($p>0.94$, unpaired t-test, 21 WT mice with 3 s LED vs 22 TG mice with 3 s LED; $p>0.12$, one-way ANOVA, 22 TG mice with 3 s LED, 11 TG mice without LED vs 9 TG mice with 30 s LED; $p>0.79$, one-way ANOVA, 22 TG mice with 3 s LED, 8 TG mice with 3 s LED and without retinal vs 18 TG mice with 3 s LED and with DPCPX).

## Supplementary Figure S7

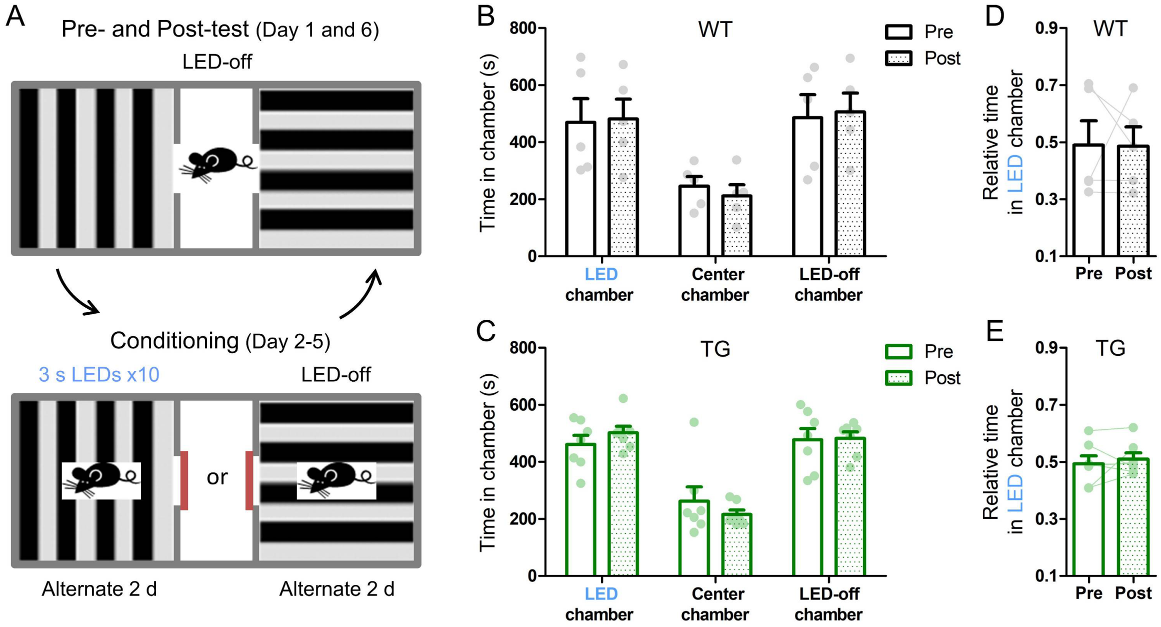


**Supplementary Figure S7**: Anterior cortical astrocytic Gq activation does not affect conditioned place preference.

(A) On day 1, mice freely entered and explored in the three chambers for 20 min to measure default place preference (pre-test). The left and right chambers were distinguished by their walls with vertical-stripes or horizontal-stripes, respectively. On day 2 to 5, mice pre-treated with retinal were confined for 30 min to either the left or right chamber on alternate days, and LED illuminations (duration 3 s, interval 3 min, 10 times) were delivered in either the left or right chamber (conditioning). Days with LED illuminations (day 2 and day 4 or day 3 and day 5) were counterbalanced. On day 6, mice again freely entered and explored in the three chambers for 20 min to measure the post-conditioning place preference (post-test).

(B) Time in each chamber was not significantly different between pre- and post-test in WT mice ($p>0.99$, two-way ANOVA, 5 WT mice).

(C) Time in each chamber was not significantly different between pre- and post-test in TG mice ($p>0.99$, two-way ANOVA, 7 strong TG mice).

(D) Relative time in LED chamber did not change between pre- and post-test in WT mice ($p>0.96$, paired t-test).

(E) Relative time in LED chamber did not change between pre- and post-test in TG mice ($p>0.55$, paired t-test).

## Supplementary Figure S8

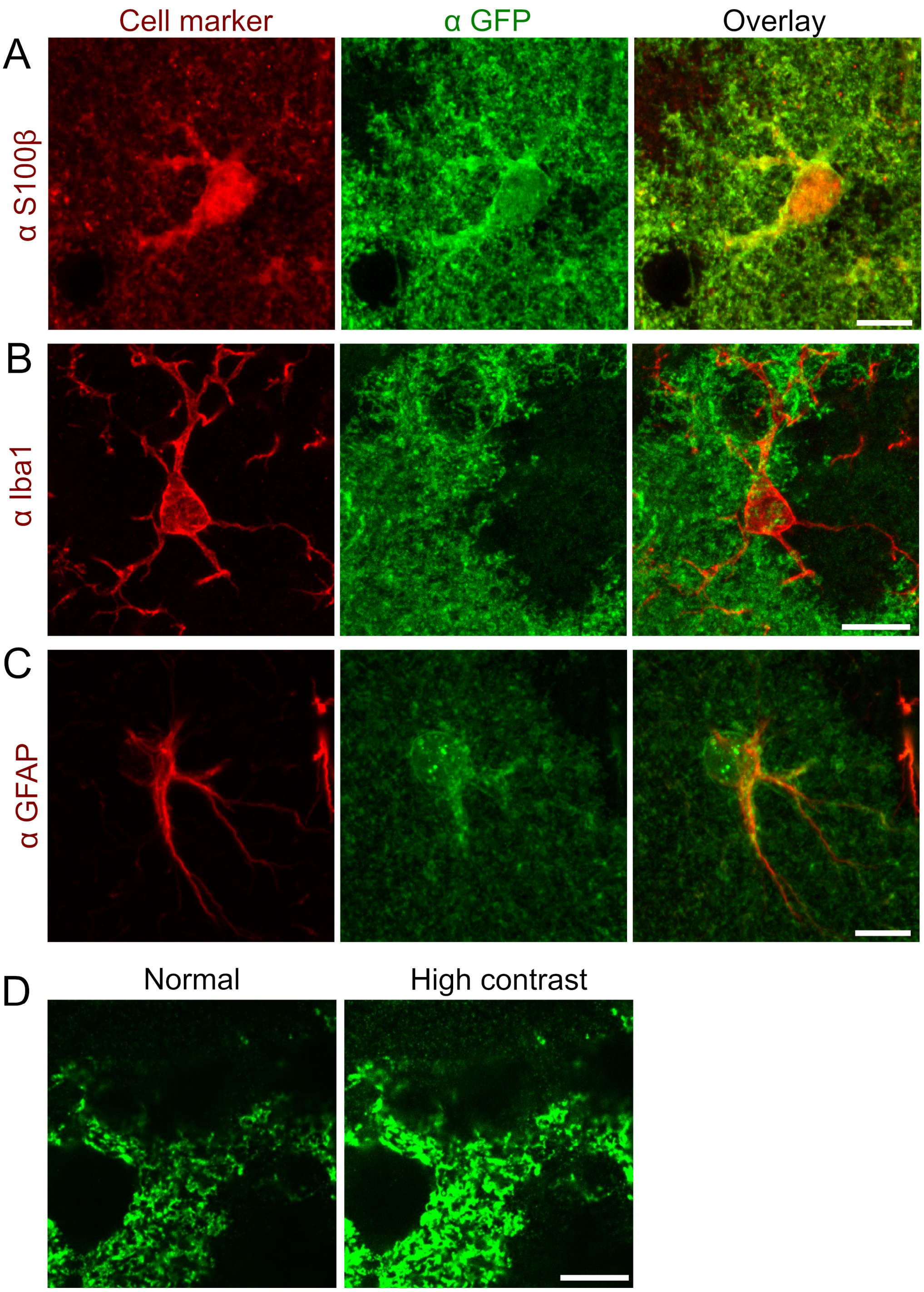


**Supplementary Figure S8**: Confocal images of the "Patchy" TG line with cell-type specific markers in the cerebral cortex.

Immunohistochemistry was performed on cerebral cortical slices with GFP antibody (green) and cell-type specific markers (red): (A) S100β for astrocytes, (B) Iba1 for microglia, (C) GFAP for astrocyte intermediate filaments. The respective samples were examined using a confocal microscope. (D) Contrast enhancement of a GFP-immunostained confocal image indicate a weak immunoreactivity in a negative patch area (upper part). Scale bars: 10 µm

## Supplementary Figure S9

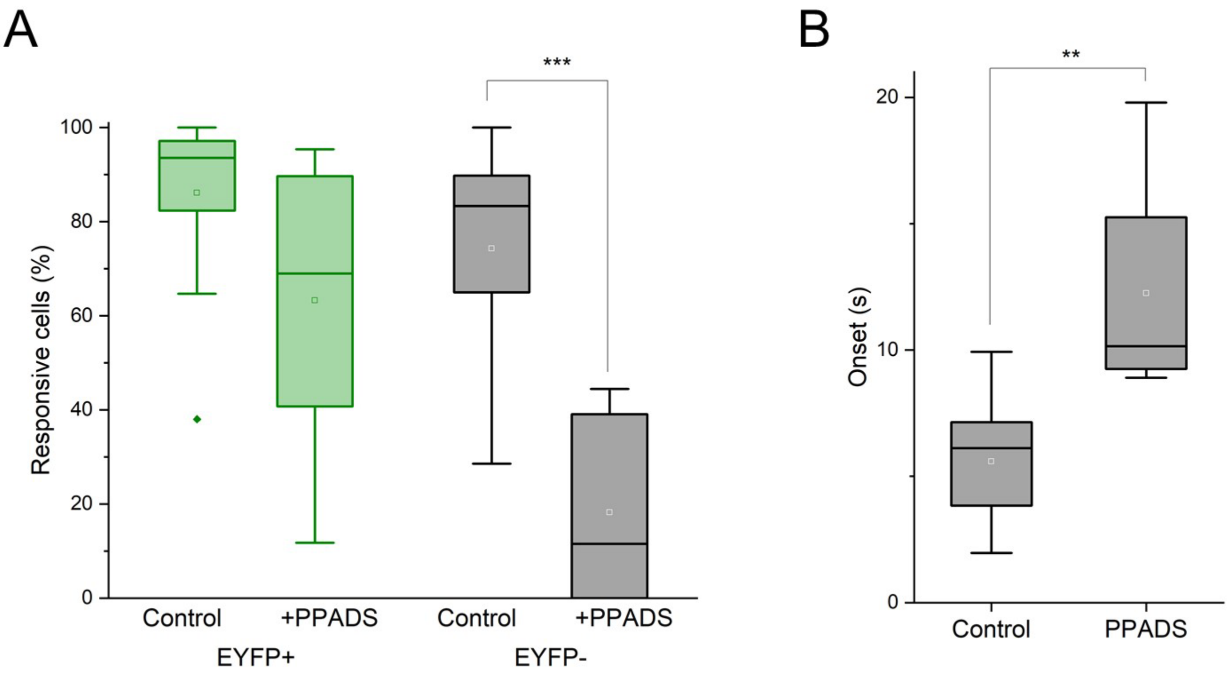


**Supplementary Figure S9**: Purinergic receptor blockade results in diminishment of delayed activation in Optoα1AR-negative astrocytes in the "Patchy" TG line.

(A) Topical application of PPADS (1 mM) results in a diminishment of activation of Optoα1AR-negative astrocytes after strong LED illumination (470 nm, 1mW, 1 s; EFYP-negative: Control vs. PPADS $p < 0.001$, t-test).

(B) The onset of $Ca^{2+}$ elevation is significantly delayed in Optoα1AR-negative astrocytes that were activated after strong LED illumination in the presence of PPADS ($p < 0.01$, Mann-Whitneytest).

## Supplementary Figure S10

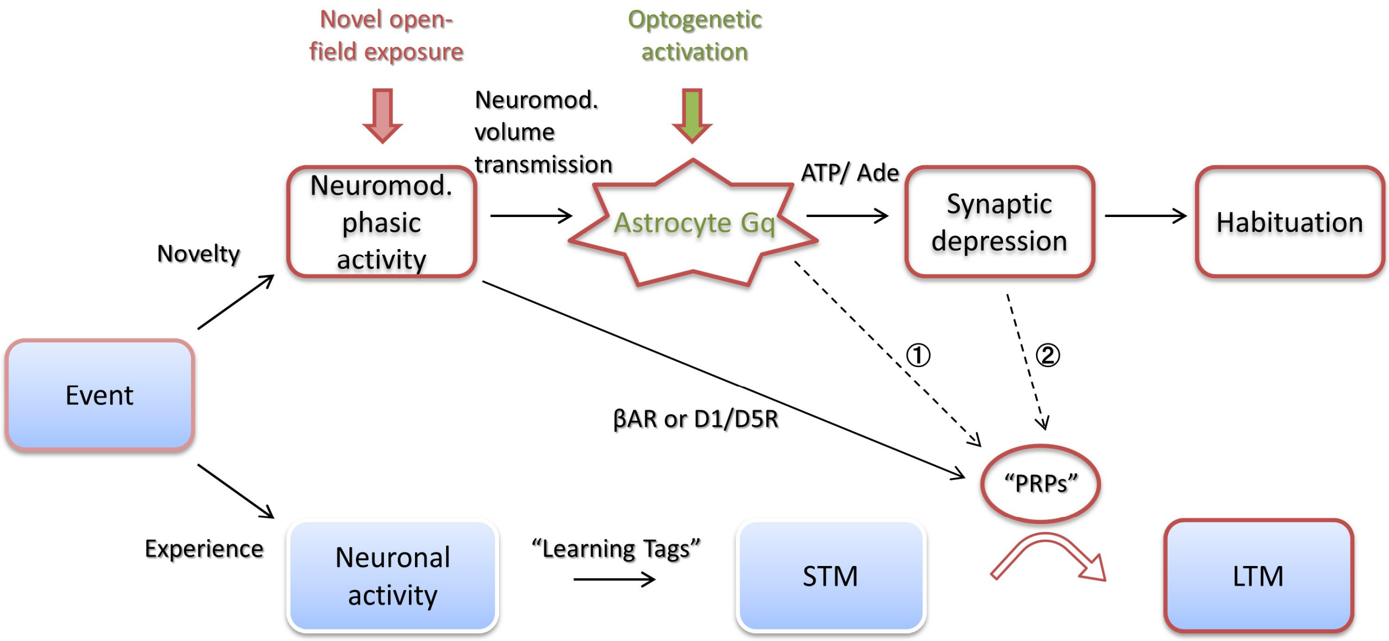


**Supplementary Figure S10**: Proposed model for astrocytic Gq signaling-mediated enhancement of long-term memory in novelty detection.

Novel experience induces synaptic depression, behavioral habituation, and LTM enhancement (all outlined with red). Our findings indicate that optogenetic activation of astrocytic Gq signaling can strengthen these novelty-induced effects through A1R signaling. Events are usually memorized only for a short time, unless they contain sufficient novelty to activate neuromodulators. When novelty is increased by novel open-field exposure, the resultant neuromodulator volume transmission including noradrenaline from the LC phasic activity converts STM to LTM, which likely involves β adrenergic receptors and D1/D5 dopaminergic receptors. According to the "synaptic and behavioral tagging" hypothesis, event-related neuronal activity sets "learning tags" in the relevant cells and synapses, and novelty-induced LC activity synthesizes "plasticity-related proteins (PRPs)", which are captured by learning tags to promote memory consolidation. Astrocytic Gq signaling induced by LC activity can contribute to the long-term memory enhancement presumably through A1R signaling-dependent PRPs synthesis (①) and/or synaptic depression-related mechanisms (②).

## Supplemental Video 1

**Supplemental Video 1**: Astrocytic $Ca^{2+}$ imaging upon brief Optoα1AR activation by weak LED illumination, corresponding to the Figure 1F.

Rhod-2 $F/F_0$ movie was overlaid on a static image of Optoα1AR-EYFP (green) and Rhod-2 (red). White circles and arrowheads at the beginning of the movie indicate EYFP-positive and negative astrocytes, respectively. Weak LED illumination (0.1 mW, 1 s, blue screen at time 0–1 s) increases $Ca^{2+}$ in EYFP-positive astrocytes, but not in EYFP-negative astrocytes. Scale bar: 50 µm; Color bar: 300 % $F/F_0$.

## Supplemental Video 2

**Supplemental Video 2**: Astrocytic $Ca^{2+}$ imaging upon brief Optoα1AR activation by strong LED illumination, corresponding to the Figure 1G.

Rhod-2 $F/F_0$ movie was overlaid on a static image of Optoα1AR-EYFP (green) and Rhod-2 (red). White circles and arrowheads at the beginning of the movie indicate EYFP-positive and negative astrocytes, respectively. Strong LED illumination (1 mW, 1 s, blue screen at time 0–1 s) increases $Ca^{2+}$ in EYFP-positive astrocytes, which appears to propagate to EYFP-negative astrocytes. Scale bar: 50 µm; Color bar: 300 % $F/F_0$.

## Supplemental Video 3

**Supplemental Video 3**: Neuronal $Ca^{2+}$ imaging upon brief Optoα1AR activation, corresponding to Figure 3A.

(Upper left) Static image of Optoα1AR-EYFP (green) and jRGECO1a (red). White rectangle indicates the analyzed area in Figure 3A. Scale bar: 100 µm.

(Upper right) jRGECO1a $F/F_0$ movie. White rectangle at the beginning of the movie indicates the same area as Figure 3A. LED illumination (1 mW, 1 s, blue screen at time 0–1 s) induces a rapid neuronal $Ca^{2+}$ decrease in the entire field of view, on which LED has been illuminated through the objective lens.

(Bottom) jRGECO1a $F/F_0$ traces of cell 1-5 indicated in Figure 3A are synchronized with the movie. Scale bars: 50 % $F/F_0$ and 15 s.
